# Knowledge-fused differential dependency network models for detecting significant rewiring in biological networks

**DOI:** 10.1186/s12918-014-0087-1

**Published:** 2014-07-24

**Authors:** Ye Tian, Bai Zhang, Eric P Hoffman, Robert Clarke, Zhen Zhang, Ie-Ming Shih, Jianhua Xuan, David M Herrington, Yue Wang

**Affiliations:** 1 Department of Electrical & Computer Engineering, Virginia Tech, Arlington 22203, VA, USA; 2 Department of Pathology, Johns Hopkins University, Baltimore 21231, MD, USA; 3 Research Center for Genetic Medicine, Children’s National Medical Center, Washington 20010, DC, USA; 4 Lombardi Comprehensive Cancer Center, Georgetown University, Washington 20057, DC, USA; 5 Department of Internal Medicine, Section on Cardiovascular Medicine, Wake Forest School of Medicine, Winston-Salem 27157, NC, USA

**Keywords:** Biological networks, Probabilistic graphical models, Differential dependency network, Network rewiring, Network analysis, Systems biology, Knowledge incorporation, Convex optimization

## Abstract

**Background:**

Modeling biological networks serves as both a major goal and an effective tool of systems biology in studying mechanisms that orchestrate the activities of gene products in cells. Biological networks are context-specific and dynamic in nature. To systematically characterize the selectively activated regulatory components and mechanisms, modeling tools must be able to effectively distinguish significant rewiring from random background fluctuations. While differential networks cannot be constructed by existing knowledge alone, novel incorporation of prior knowledge into data-driven approaches can improve the robustness and biological relevance of network inference. However, the major unresolved roadblocks include: big solution space but a small sample size; highly complex networks; imperfect prior knowledge; missing significance assessment; and heuristic structural parameter learning.

**Results:**

To address these challenges, we formulated the inference of differential dependency networks that incorporate both conditional data and prior knowledge as a convex optimization problem, and developed an efficient learning algorithm to jointly infer the conserved biological network and the significant rewiring across different conditions. We used a novel sampling scheme to estimate the expected error rate due to “random” knowledge. Based on that scheme, we developed a strategy that fully exploits the benefit of this data-knowledge integrated approach. We demonstrated and validated the principle and performance of our method using synthetic datasets. We then applied our method to yeast cell line and breast cancer microarray data and obtained biologically plausible results. The open-source R software package and the experimental data are freely available at http://www.cbil.ece.vt.edu/software.htm.

**Conclusions:**

Experiments on both synthetic and real data demonstrate the effectiveness of the knowledge-fused differential dependency network in revealing the statistically significant rewiring in biological networks. The method efficiently leverages data-driven evidence and existing biological knowledge while remaining robust to the false positive edges in the prior knowledge. The identified network rewiring events are supported by previous studies in the literature and also provide new mechanistic insight into the biological systems. We expect the knowledge-fused differential dependency network analysis, together with the open-source R package, to be an important and useful bioinformatics tool in biological network analyses.

## Background

Biological networks are context‐specific and dynamic in nature [1]. Under different conditions, different regulatory components and mechanisms are selectively activated or deactivated [2],[3]. One example is the topology of underlying biological network changes in response to internal or external stimuli, where cellular components exert their functions through interactions with other molecular components [4],[5]. Thus, in addition to asking “which genes are differentially expressed”, the new question is “which genes are differentially connected” [6],[7]. Studies on network-altering events will shed new light on whether network rewiring is a general principle of biological systems regarding disease progression or therapeutic responses [2],[3]. Moreover, due to inevitable experimental noise, snapshots of dynamic expression, and post-transcriptional or translational/post-translational modifications, systematic efforts to characterize biological networks must effectively distinguish significant network rewiring from random background fluctuations [1].

Almost exclusively using high-throughput gene expression data and focusing on conserved biological networks, various network inference approaches have been proposed and tested [1], including probabilistic Boolean networks [8], state‐space models [9],[10], and probabilistic graphical models [11]. However, since these methods often assume that there is a static network structure, they overlook the inherently dynamic nature of molecular interactions, which can be extensively rewired across different conditions. Hence, current network models only present a conserved cellular network averaging across all samples. To explicitly address differential network analysis [3],[5],[12], some initial efforts have been recently reported [1]. In our previous work, Zhang et al. proposed to model differential dependency networks between two conditions by detecting network rewiring using significance tests on local dependencies across conditions [13],[14], which is a substantially different method from the one proposed in this paper where experimental data and prior knowledge are jointly modeled. The approach was successfully extended by Roy et al. to learn dynamic networks across multiple conditions [15], and by Gill et al. to assess the overall evidence of network differences between two conditions using the connectivity scores associated with a gene or module [16]. Pioneered and reported in [17], correlation and partial correlation are used to construct network graphs, and differential pathway analysis is developed based on graph edit distance. The temporal evolution of network structures is examined with a fused penalty term to encode relationship between adjacent time points in [18]. Furthermore, recent efforts have also been made to incorporate existing knowledge about network biology into data-driven network inference [19]. Wang et al. proposed to incorporate prior knowledge into the inference of conserved networks in a single condition by adjusting the Lasso penalties [20]. Yet, the inherently dynamic wiring of biological networks remains under-explored at the systems level, as interaction data are typically reported under diverse and isolated conditions [1].

There are at least five unresolved issues concerning differential network inference using data-knowledge integrated approaches: (1) the solution (search) space is usually large while sample sizes are small, resulting in potential overfitting; (2) both conserved and differential biological networks are complex and lack closed-form or efficient numerical solutions; (3) “structural” model parameters are assigned heuristically, leading to potentially suboptimal solutions; (4) prior knowledge is imperfect for inferring biological networks under specific conditions, e.g., false positive “connections”, biases, and non-specificity; and (5) most current methods do not provide significance assessment on the differential connections and rigorous testing of the type I error rate.

To address these challenges, we formulated the inference of differential dependency networks that incorporate both conditional data and prior knowledge as a convex optimization problem, and developed an efficient learning algorithm to jointly infer the conserved biological network and the significant rewiring across different conditions. Extending and improving our work on Gaussian graphical models [21],[22], we designed block-wise separable penalties in the Lasso-type models that permit joint learning and knowledge incorporation with an efficient closed-form solution. We estimated the expected error rate due to “random” prior knowledge via a novel sampling scheme. Based on that scheme, we developed a strategy to fully exploit the benefit of this data-knowledge integrated approach. We determined the values of model parameters that quantitatively correspond to the expected significance level, and evaluated the statistical significance of each of the detected differential connections. We validated our method using synthetic datasets and comprehensive comparisons. We then applied our method to yeast cell line and breast cancer microarray data and obtained biologically plausible results.

## Methods

### Formulation of knowledge-fused differential dependency network (kDDN)

We represent the condition‐specific biological networks as graphs. Suppose there are *p* nodes (genes) in the network of interest, and we denote the vertex set as *V*. Let *G*^(1)^ = (*V*, *E*^(1)^) and *G*^(2)^ = (*V*, *E*^(2)^) be the two undirected graphs under the two conditions. *G*^(1)^ and *G*^(2)^ have the same vertex set *V*, and condition‐specific edge sets *E*^(1)^ and *E*^(2)^. The edge changes indicated by the differences between *E*^(1)^ and *E*^(2)^ are of particular interest, since such rewiring may reveal pivotal information on how the organisms respond to different conditions. We label the edges as common edges or specific to a particular condition in graph *G* = (*V*, *E*) to represent the learned networks under the two conditions.

Prior knowledge on biological networks is obtained from well-established databases such as KEGG [19] and is represented as a knowledge graph *G*_**W**_ = (*V*, *E*_**W**_), where the vertex set *V* is the same set of nodes (genes) and the edge set *E*_W_ over *V* is translated from prior knowledge. There are many alternatives to extract existing domain knowledge, e.g., STRING, HPRD, or manual construction. The adjacency matrix of *G*_W_, **W** ∈ *ℜ*^*p* × *p*^, is used to encode the prior knowledge. The elements of **W** are either 1 or 0, with *X*_*ji*_ = 1 indicating the existence of an edge from the *j*^th^ gene to the *i*^th^ gene (or their gene products), where *i*, *j* = 1, 2, ⋯, *p*, *i* ≠ *j*. **W** is symmetric if the prior knowledge is not directed.

**Figure 1:**
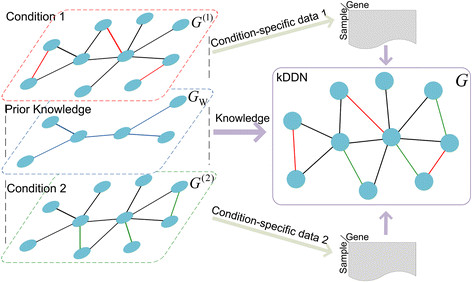
**Knowledge-fused differential dependency network learning.** The algorithm takes condition-specific data and prior knowledge as input and infers condition-specific networks. Black edges are common edges. Red and green edges are differential edges specific to conditions.

The main task in this paper is to infer from data and prior knowledge *G*_W_ the condition‐specific edge sets *E* (both *E*^(1)^ and *E*^(2)^). The method is illustrated in Figure 1.

We consider the *p* nodes in *V* as *p* random variables, and denote them as *X*_1_, *X*_2_, ⋯, *X*_*p*_. Suppose there are *N*_1_ samples under condition 1 and *N*_2_ samples under condition 2. Without loss of generality, we assume *N*_1_ = *N*_2_ = *N*. Under the first condition, for variable X_*i*_, we have observations $x_{i}^{\left(1\right)}={\left[x_{1i}^{\left(1\right)},x_{2i}^{\left(1\right)},\cdots ,x_{\mathrm{Ni}}^{\left(1\right)}\right]}^{T}$, while under the second condition, we have $x_{i}^{\left(2\right)}={\left[x_{1i}^{\left(2\right)},x_{2i}^{\left(2\right)},\cdots ,x_{\mathrm{Ni}}^{\left(2\right)}\right]}^{T}$, i = 1,2,⋅⋅⋅,*p*. Further, let $X^{\left(1\right)}=\left[x_{1}^{\left(1\right)},x_{2}^{\left(1\right)},\cdots ,x_{p}^{\left(1\right)}\right]$ be the data matrix under condition 1 and $X^{\left(2\right)}=\left[x_{1}^{\left(2\right)},x_{2}^{\left(2\right)},\cdots ,x_{p}^{\left(2\right)}\right]$ be the data matrix under condition 2.

Denote

(1) $$y_{i}=\left[\begin{array}{c} x_{i}^{\left(1\right)}\\ x_{i}^{\left(2\right)} \end{array}\right],\;X=\left[\begin{array}{cc} X^{\left(1\right)} & 0\\ 0 & X^{\left(2\right)} \end{array}\right],$$

and

(2) $$\beta_{i}=\left[\begin{array}{c} \beta_{i}^{\left(1\right)}\\ \beta_{i}^{\left(2\right)} \end{array}\right]={\left[\beta_{1i}^{\left(1\right)},\beta_{2i}^{\left(1\right)},\cdots ,\beta_{\mathrm{pi}}^{\left(1\right)},\beta_{1i}^{\left(2\right)},\beta_{2i}^{\left(2\right)},\cdots ,\beta_{\mathrm{pi}}^{\left(2\right)}\right]}^{T},$$

with the non‐zero elements of $\beta_{i}^{\left(1\right)}$ indicating the neighbours of the *i*^th^ node under the first condition and the non‐zero elements of $\beta_{i}^{\left(2\right)}$ indicating the neighbours of the *i*^th^ node under the second condition.

The problem of simultaneously learning network structures and their changes under two conditions is formulated as a regularized linear regression problem with sparse constraints and solved by convex optimization. For each node (variable) *X*_*i*_, i = 1,2,⋅⋅⋅,*p*, we solve the optimization with the objective function

(3) $$f\left(\beta_{i}\right)=\frac{1}{2}{\left\|y_{i}-X\beta_{i}\right\|}_{2}^{2}+\lambda_{1}\sum_{j=1}^{P}\left(1-W_{\mathrm{ji}}\theta \right)\left(\left|\beta_{\mathrm{ji}}^{\left(1\right)}\right|+\left|\beta_{\mathrm{ji}}^{\left(2\right)}\right|\right)+\lambda_{2}{\left\|\beta_{i}^{\left(1\right)}-\beta_{i}^{\left(2\right)}\right\|}_{1}$$

The non‐zero elements in **W** introduce knowledge to the objective function (3), and *θ* is a *ℓ*_1_ penalty relaxation parameter taking value in [0, 1].

The solution is obtained by minimizing (3),

(4) $$\begin{array}{l} \beta_{i}=\arg\min_{\beta_{i}}f\left(\beta_{i}\right)\\ \;=\arg\min_{\beta_{i}^{\left(1\right)},\beta_{i}^{\left(2\right)}}\frac{1}{2}{\left\|y_{i}-X\beta_{i}\right\|}_{2}^{2}+\lambda_{1}\sum_{j=1}^{p}\left(1-W_{\mathrm{ji}}\theta \right)\left(\left|\beta_{\mathrm{ji}}^{\left(1\right)}|+|\beta_{\mathrm{ji}}^{\left(2\right)}\right|\right)+\lambda_{2}{\left\|\beta_{i}^{\left(1\right)}-\beta_{i}^{\left(2\right)}\right\|}_{1}\\ \;s.t.\;\beta_{\mathrm{ii}}^{\left(1\right)}=0,\beta_{\mathrm{ii}}^{\left(2\right)}=0. \end{array}$$

Both the cost function $\frac{1}{2}{\left\|y_{i}-X\beta_{i}\right\|}_{2}^{2}$ and two regularization terms $\sum_{j=1}^{p}\left(1-W_{\mathrm{ji}}\theta \right)\left(\left|\beta_{\mathrm{ji}}^{\left(1\right)}|+|\beta_{\mathrm{ji}}^{\left(2\right)}\right|\right)$ and $\left\|\beta_{i}^{\left(1\right)}-\beta_{i}^{\left(2\right)}\right\|$ co-existed in the objective function are convex, and this convex formulation leads to an efficient algorithm. The structures of the graphical model under two conditions are obtained jointly by solving (4) sequentially for all nodes. The inconsistency between $\beta_{i}^{\left(1\right)}$ and $\beta_{i}^{\left(2\right)}$ highlights the structural changes between two conditions, and the collection of differential edges form the differential dependency network.

Given the vast search space and complexity in both conserved and differential networks, it is crucial for kDDN to identify statistically significant network changes and filter the structural and parametric inconsistencies due to noise in the data and limited samples. This objective is achieved by selecting the proper model specified by *λ*_1_ and *λ*_2_ that best fits the data and suffices the statistical significance. *λ*_1_ is determined by controlling the probability of falsely joining two distinct connectivity components of the graph [23] and *λ*_2_ is found by setting differential edges to a defined significance level. We refer readers to Additional file 1: S4.1 for a detailed discussion of model parameter-setting approaches.

With parameters specified, problem (4) can be solved efficiently by the block coordinate descent algorithm presented in Additional file 1: S4.3, Algorithm S1.

### Incorporation of prior knowledge

The prior knowledge is explicitly incorporated into the formulation by *W*_*ji*_ and *θ* in the block-wise weighted *ℓ*_1_ -regularization term. *W*_*ji*_ = 1 indicates that the prior knowledge supports an edge from the *j*^th^ gene to the *i*^th^ gene and 0 otherwise. A proper *θ* will reduce the penalty applied to $\beta_{\mathrm{ji}}^{\left(c\right)}$, c = 1, 2, corresponding to the connection between *X*_*j*_ and *X*_*i*_ with *W*_*ji*_ = 1. As a result, the connection between *X*_*j*_ and *X*_*i*_ will more likely be detected.

*θ* is a weighting parameter on the influence of prior knowledge, determining the degree of the knowledge incorporation in the network inference. When *θ* = 0, the algorithm ignores all knowledge information and gives solely data-based results; conversely, when *θ* = 1, the edge between *X*_*j*_ and *X*_*i*_ will always be included if such an edge exists in the prior knowledge. Therefore, the prior knowledge incorporation needs to find a proper balance between the experimental data and prior knowledge to achieve effective incorporation, as well as limit the adverse effects caused by any spurious edges contained in imperfect prior knowledge.

Here we propose a strategy to control the adverse effects incurred in the worst‐case scenario under which the given prior knowledge is totally random. In this case, the entropy of the knowledge distribution over the edges is maximized and the information introduced to the inference is minimal. Incorporating such random knowledge, the inference results will deviate from the purely data-driven result. We want to maximize the incorporation of relevant prior knowledge, while at the same time making sure the potential negative influence of irrelevant prior knowledge is under control so that the expected deviation is confined within an acceptable range in the worst‐case scenario. To properly set the value of *θ*, we assess the actual influence of prior knowledge for each value that *θ* may take, and developed Theorem 1 to determine the best degree of prior knowledge incorporation. This approach guarantees robustness even when the prior knowledge is highly inconsistent with the underlying ground truth.

To quantify the effects of prior knowledge incorporation, we use graph edit distance [24] between two adjacency matrices as a measurement for the dissimilarity of two graphs. Let *G*_T_ = (*V*, *E*_T_) denote the ground‐truth graph with edge set *E*_*T*_, *G*_X_ = (*V*, *E*_X_) denote the graph learned purely from data, i.e., **W** = **0**, and $G_{X,W_{R},\theta }=\left(V,E_{X,W_{R},\theta }\right)$ denote the graph learned with prior knowledge. **W**_R_ indicates that the prior knowledge is “random”. Let *d*(*G*_1_, *G*_2_) denote the graph edit distance between two graphs. Further, let |*E*| be the number of edges in the graph *G*.

Our objective is to bound the increase of inference error rate associated with the purely data-driven result, $d\left(G_{T},G_{X,W_{R},\theta }\right)/\left|E_{T}\right|-d\left(G_{T},G_{X}\right)/\left|E_{T}\right|$, within an acceptable range *δ* even if the prior knowledge is the worst case by finding a proper *θ*.

Since *G*_T_ is unknown, we instead control the increase in the error rate indirectly by evaluating the effect of random knowledge against *G*_X_, the purely data‐driven inference result. Specifically, we use a sampling‐based algorithm to find the empirical distribution of $d\left(G_{X},G_{X,W_{R},\theta }\right)$, and choose the largest *θ* ∈ [0, 1] that satisfies:

(5) $$\begin{array}{l} \hat{\theta }=\mathrm{max\theta}\\ s.t.\;E\left[d\left(G_{X},G_{X,W_{R},\theta }\right)\right]/\left|E_{X}\right|\leq \delta , \end{array}$$

where *E*[*d*(*G*_1_, *G*_2_)] is the expectation of the graph edit distance between graphs *G*_1_ and *G*_2_, with respect to its empirical distribution.

A natural question is whether using *G*_X_ instead of *G*_T_ to control the increase in the error rate induced by random knowledge is legitimate. To answer this question, we show in Theorem 1 (proof included in Additional file 1: S2) that the *θ* obtained in (5) in fact controls an upper bound of $E\left[d\left(G_{T},G_{X,W_{R},\theta }\right)\right]/\left|E_{T}\right|$, i.e. the increase in the network inference error rate induced by random prior knowledge (the worst‐case scenario), under the assumption that the number of false negatives (*FN*) in the data-driven result *G*_X_ is smaller than the number of false positives (*FP*). As we adopt a strategy to control the probability of falsely joining two distinct connectivity components [23], this assumption generally holds.

Theorem 1 establishes the relationship between prior knowledge incorporation *θ* and the adverse effects of prior knowledge on network inference, quantified by *δ*, under the worst-case scenario (when the prior knowledge is completely irrelevant). For example, *δ* = 0.1 indicates that the user can accept at most 10% performance degradation if the prior knowledge is completely noise. With the estimate of *θ* at *δ* = 0.1, even the prior knowledge is totally random, the performance will decrease no more than 10%, while the relevant portion of the real prior knowledge (better than random noise) can greatly improve the overall network inference performance.

#### Theorem 1

*For a given δ* ∈ [0, 1)*, if the prior knowledge incorporation parameter θ satisfies the inequality*

(6) $$\frac{E\left[d\left(G_{X},G_{X,W_{R},\theta }\right)\right]}{\left|E_{X}\right|}\leq \delta ,$$

*then the increase in the error rate induced by incorporating random prior knowledge is bounded by δ, more specifically,*

(7) $$\frac{E\left[d\left(G_{T},G_{X,W_{R},\theta }\right)\right]}{\left|E_{T}\right|}\leq \frac{d\left(G_{T},G_{X}\right)}{\left|E_{T}\right|}+\delta$$

Given the number of edges specified in the prior knowledge, procedures to compute *θ* are detailed in Algorithm S2 in Additional file 1: S4.3.

## Results and discussion

We demonstrated the utility of kDDN using both simulation data and real biological data. In the simulation study, we tested our method on networks with different sizes and compared with peer methods the performance of overall network structure recovery, differential network identification and tolerance of false positives in the prior knowledge.

In a real data application, we used kDDN to learn the network rewiring of the cell cycle pathway of budding yeast in response to oxidative stress. A second real data application was the study of the differential apoptotic signaling between recurring and non-recurring breast cancer tumors. Applications to study muscular dystrophy and transcription factor binding schemes are included in Additional file 1: S6.

### Simulation study

We constructed a Gaussian Markov random field with *p* = 100 nodes and 150 samples following the approach used in [23], and then randomly modified 10% of the edges to create two condition‐specific networks with sparse changes. The details of simulation data generation procedure are provided in Additional file 1: S5.1. The number of edges in prior knowledge *M* was set to be the number of common edges in the two condition‐specific networks, and *δ* was set to 0.1.

To assess the effectiveness of prior knowledge incorporation and robustness of kDDN when false positive edges were present in prior knowledge, we examined the network inference precision and recall of the overall network and the differential network at different levels of false positive rate in the prior knowledge.

Both false positives and false negatives in the prior knowledge here are with respect to the condition-specific ground truth from which the data are generated. Thus, although false positives in prior knowledge may contribute more learning errors, false negatives will not worsen network learning performance (results shown in Additional file 1: S5.5).

Starting from prior knowledge without any false positive edges, we gradually increased the false positive rate in prior knowledge until all prior knowledge was false. At each given false positive rate in the prior knowledge, we randomly created 1,000 sets of prior knowledge, and compared the performance of kDDN in terms of precision and recall with two baselines: (1) a purely data-driven result, corresponding to kDDN with *θ*=0, i.e., without using any prior knowledge in the network inference (using only data for network inference); and (2) a naïve baseline of knowledge incorporation by superimposing (union) the prior knowledge network and the network inferred purely from the data.

**Figure 2:**
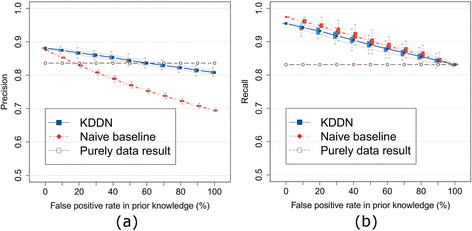
**Effects of false positive rates in prior knowledge on inference of the overall network. (a)** Precision of overall network inference. **(b)** Recall of overall network inference. The experiments show that true knowledge improves both precision and recall of overall network inference, and the maximum degradation of inference results is bounded when the prior knowledge is imperfect.

The results of the overall network (both common and differential edges) learning are shown in Figure 2(a) and (b). The dot-connected lines are averaged precision or recall of the network learned with 1,000 sets of prior knowledge. The box plot shows the first, second and third quartiles of precision or recall at each false positive rate in prior knowledge (with the ends of the whiskers extending to the lowest datum within a 1.5 interquartile range of the lower quartile, and the highest datum within a 1.5 interquartile range of the upper quartile). The blue squared lines, brown circle lines, and red diamond lines indicate the mean performance of kDDN, purely data-driven baseline, and naïve baseline, respectively. Narrower lines with the same colors and line styles mark the one standard deviation of the performance of the corresponding approach.

Precision reflects the robustness to the false positive edges and efficiency of utilizing the information in prior knowledge. Figure 2(a) shows that, as expected, the false positive rate in prior knowledge has a limited effect on the precision of kDDN (blue squared lines). With more false positives in the prior knowledge, the precision decreases but is still around the purely data-driven baseline (brown circle lines) and much better than the naïve baseline (red diamond lines). The naïve baseline suffers significantly from the false positives in prior knowledge, because it indiscriminately accepts all edges in prior knowledge without considering evidence in the data. This observation corroborates the design of our method: to control the false detection incurred by the false positives in the prior knowledge. At the point where the false positive rate in the prior knowledge is 100%, the decrease of precision compared with the purely data-based result is bounded within *δ*.

Recall reflects the ability of prior knowledge in helping recover missing edges. Figure 2(b) shows that when the prior knowledge is 100% false, the recall is the same as that of the purely data-driven result, because in this case the prior knowledge brings in no useful information for correct edge detection. When the true positive edges are included in the prior knowledge, the recall becomes higher than that of the purely data-based result, because more edges are correctly detected by harnessing the correct information in the prior knowledge. The naïve baseline is slightly higher in recall, since it calls an edge as long as knowledge contains it, while kDDN calls an edge only when both knowledge and data evidence are present. The closeness between kDDN and naïve baseline demonstrates the high efficiency of our method in utilizing the true information in prior knowledge.

**Figure 3:**
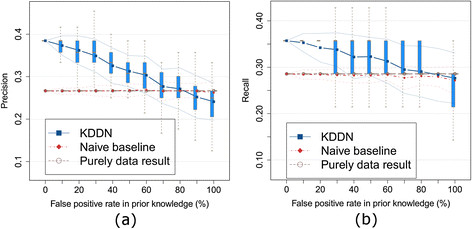
**Effects of false positive rates in prior knowledge on inference of the differential network. (a)** Precision of differential network inference. **(b)** Recall of differential network inference. The experiments show that true knowledge improves both precision and recall of differential network inference. The maximum degradation of inference results is bounded when the prior knowledge is imperfect.

We then evaluated the effect of knowledge incorporation solely on the identification of differential networks following the same protocol. The results are shown in Figure 3 with the same color and line annotations.

For differential network recovery, the naïve baseline is almost identical to purely data-driven results because the prior knowledge seldom includes a differential edge in a large network with sparse changes. While similar advantages of kDDN apply, our method has better precision and recall, and bounds the performance degradation when knowledge is totally wrong. Unlike the naïve baseline where knowledge and data are not linked, we model the inference with knowledge and data together, so knowledge is also able to help identify differential edges. Performance evaluation results in Additional file 1: S5.3 studied networks with varying sizes, reaching consistent conclusions.

Depending on specific conditions, false positives in prior knowledge may not distribute uniformly, but tend to aggregate more towards certain nodes. Experiments with biased knowledge distribution shown in Additional file 1: S5.4, Figures S10-S13 indicate no difference or little improvement compared to random knowledge, confirming that random knowledge represents the worst-case scenario and the bound according to random knowledge is sufficient.

### Performance comparisons with peer methods

We compared our joint learning method kDDN with four peer methods: 1) DDN (independent learning) [13], 2) csLearner (joint learning) [15], 3) Meinshausen’s method (independent learning) [23], and 4) Tesla (joint learning) [18]. csLearner can learn more than two networks but we restricted the condition to two. Meinshausen’s method learns the network under a single condition, and we combined the results learned under each condition to get conserved network and differential network. Tesla learns a time-evolving network, but can be adapted to two-condition learning as well. Only kDDN can assign edge-specific *p*-values to differential edges.

Parameters in kDDN are automatically inferred from data as described in Additional file 1: S4.1. For the competing methods in the comparison, we manually tested and tuned their parameters to obtain their best performance. We set DDN to detect pair-wise dependencies. The number of neighbors in csLearner is set to “4” (the ground truth value). Meinshausen’s method uses the same *λ*_1_ as inferred by kDDN, as it is a special case of kDDN under one condition without prior knowledge. Tesla uses the empirically-determined optimal parameter values, since the parameter selection was not automatic but relies on user input.

To assess the impact of prior knowledge, we ran kDDN under three scenarios: data-only (kDDN.dt), data plus true prior knowledge (kDDN.tk), and data plus “random” prior knowledge (kDDN.fk). Only kDDN is able to utilize prior knowledge.

The ground truth networks consisted of 80, 100, 120, 140 and 160 nodes, respectively, and correspondingly 120, 150, 200, 200 and 240 samples. For each network size, 100 replicate simulation networks were generated. We evaluated the performance of inferring both overall and differential edges of the underlying networks using the F-score (harmonic mean of precision and recall, $2\frac{\mathrm{precision}*\mathrm{recall}}{\mathrm{precision}+\mathrm{recall}}$) and precision-recall averaged over all datasets under each network size.

**Figure 4:**
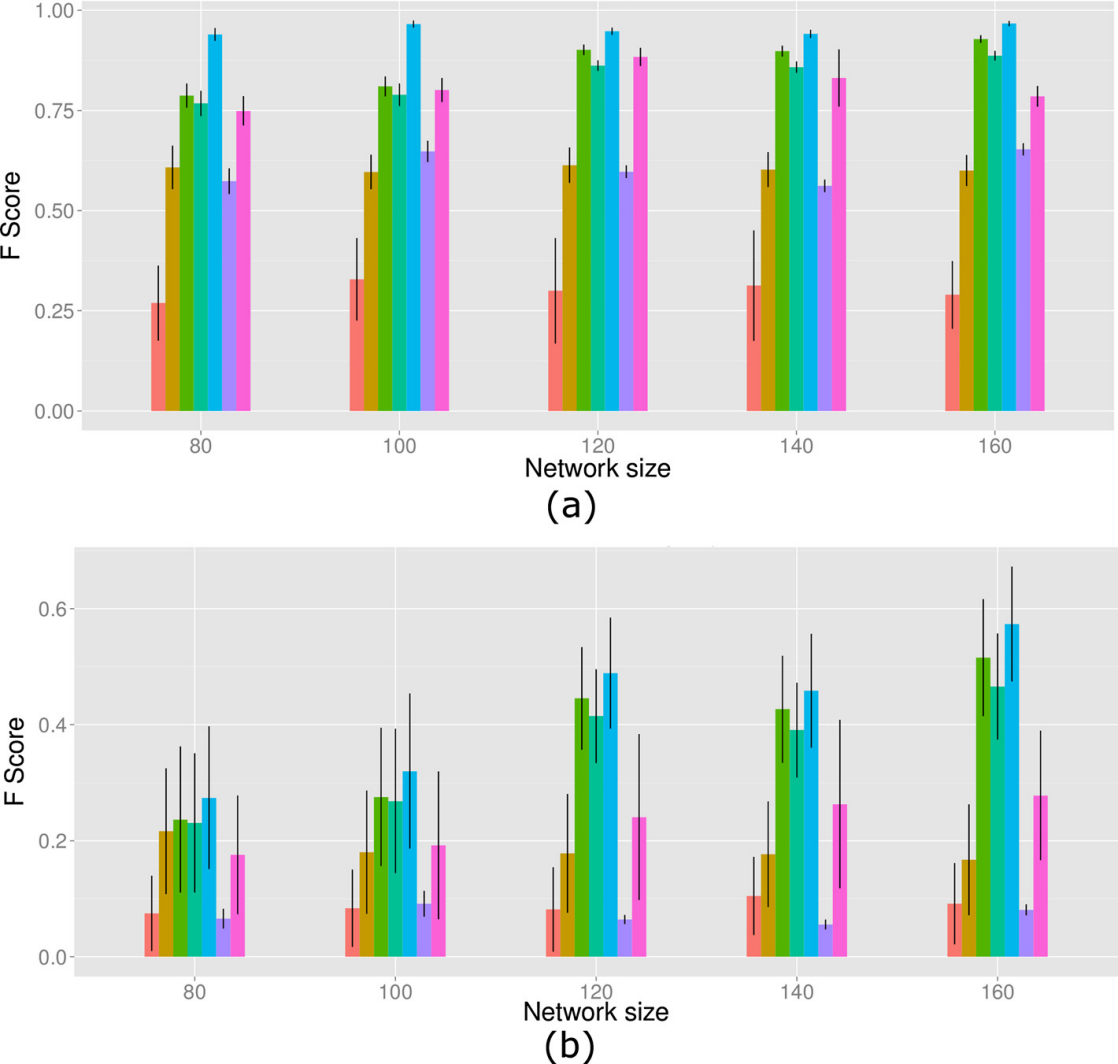
**Performance comparison in F score. (a)** Inference of overall network. **(b)** Inference of differential network. Legend (from left to right): orange-csLearner, golden-DDN, olive green-kDDN.dt, aquamarine-kDDN.fk, blue-kDDN.tk, purple-Meinshausen, magenta-Tesla. The experimental results show that kDDN outperforms the peer methods in both overall network learning and differential network inference.

The results are presented in Figure 4 using bar plots. The color annotations are: orange-csLearner, golden-DDN, olive green-kDDN.dt, aquamarine-kDDN.fk, blue-kDDN.tk, purple-Meinshausen, magenta-Tesla. We used one-sided *t*-tests to assess whether kDDN performs better than the peer methods across all network sizes. The null hypothesis is that there is no difference between the mean of F-score of kDDN and the peer methods. The alternative hypothesis is that kDDN has the greater mean of F-score. The detailed results are included in Tables S1 and S2 in Additional file 1: S5.7, which shows that kDDN.tk performs significantly better than peers in all cases, and kDDN.dt and kDDN.fk performs better than peers in 118 of 120 cases.

Figure 4(a) compares the ability of recovering overall networks. We see kDDN.tk consistently outperforms all peer methods, and kDDN.dt and kDDN.fk performs comparably to Tesla (best-performing peer method). The independent learning methods, DDN and Meinshausen’s method, place third due to their inability to jointly use data.

Figure 4(b) shows the comparison of performance on recovering differential edges. kDDN consistently outperforms all peer methods under all scenarios. While kDDN and Tesla share some commonalities, they use different formulations. Where Tesla uses logistic regression, kDDN adopted linear regression to model the dependency. Such a difference also has implications for the subsequent solutions and outcomes. The fact that kDDN determines *λ*_2_ according to the statistical significance of differential edges helps kDDN outperform Tesla in differential edge detection. It is also clear that a single-condition method cannot find the differential edges correctly and has the worst performance.

In Figures S17 and S18 in Additional file 1: S5.7, the performance of these methods is compared in terms of precision and recall; we reached the same conclusions.

Through these comparisons, we show that kDDN performs better than peer methods in both overall and differential network learning. High-quality knowledge further improves kDDN performance, while kDDN is robust enough even to totally random prior knowledge. Joint learning, utilization of prior knowledge, and attention to statistical significance all helped kDDN outperform the other methods.

### Pathway rewiring in yeast uncovers cell cycle response to oxidative stress

To test the utility of kDDN using real biological data, we applied the kDDN to the public data set GSE7645. This data set used budding yeast *Saccharomyces cerevisiae* to study the genome-wide response to oxidative stress imposed by cumene hydroperoxide (CHP). Yeast cultures were grown in controlled batch conditions, in 1L fermentors. Three replicate cultures in mid-exponential phase were exposed to 0.19mM CHP, while three non-treated cultures were used as controls. Samples were collected at t = 0 (immediately before adding CHP) and at 3,6,12,20,40,70 and 120 min after adding the oxidant. Samples were processed for RNA extraction and profiled using Affymetrix Yeast Genome S98 arrays. There were 48 samples in total, evenly divided between the treated and the non-treated groups.

**Figure 5:**
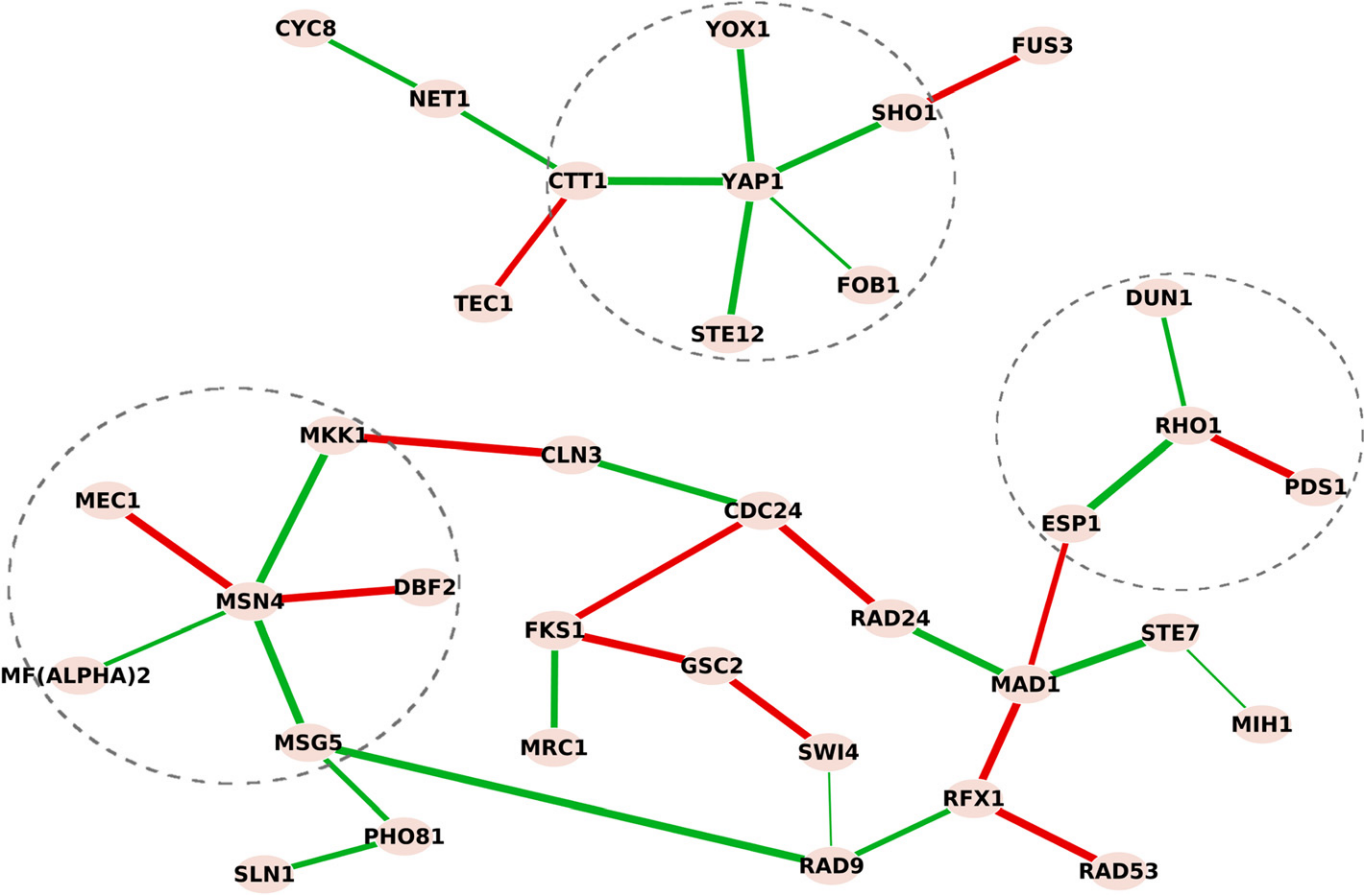
**Differential dependency network in budding yeast reflects the cell cycle response to oxidative stress.** Red edges are connections in control and green edges are connections under stress, mutually and exclusively. *Yap1*, *Rho1* and *Msn4*, the three known responders to stress response, are at the center of the inferred networks in response to oxidative stress. They are activated under oxidative stress and many connections surrounding them are activated (green edges). Acting as an antioxidant in response to oxidative stress, *Ctt1* coordinate with *Yap1* to protect cells from oxidative stress.

We analyzed the network changes of cell cycle-related genes with structural information from the KEGG yeast pathway as prior knowledge. We added the well-studied yeast oxidative stress response gene *Yap1*[25]-[28] to the knowledge network and related connections gathered from the *Saccharomyces* Genome Database [29]. The learned differential network result is shown in Figure 5, in which nodes represent genes involved in the pathway rewiring, and edges show the condition-specific connections. Red edges are connections in control and green edges are connections under stress.

Oxidative stress is a harmful condition in cells, due to the failure of the antioxidant defense system to effectively remove reactive oxygen molecules and other oxidants. The result shows that *Yap1*, *Rho1* and *Msn4* are at the center of the network response to oxidative stress; they are activated under oxidative stress and many connections surrounding them are created (green edges). *Yap1* is a major transcription factor that responds to oxidative stress [25]-[28]. *Msn4* is considered as a general responder to environmental stresses including heat shock, hydrogen peroxide, hyper-osmotic shock, and amino acid starvation [30],[31]. *Rho1* is known to resist oxidative damage and facilitate cell survival [32]-[34]. The involvement of these central genes captured the dynamic response of how yeast cells sense and react to oxidative stress. The edge between *Yap1* and *Ctt1* under stress grants more confidence to the result. *Ctt1* acts as an antioxidant in response to oxidative stress [35], and the coordination between *Yap1* and *Ctt1* in protecting cells from oxidative stress is well established [36]. This result depicted the dynamic response of yeast when exposed to oxidative stress and many predictions are supported by previous studies. This real data study validated the effectiveness of the methods in revealing underlying mechanisms and providing potentially novel insights. These insights would be largely missed by conventional differential expression analysis as the important genes *Rho1*, *Msn4*, *Yap1* and *Ctt1* ranks 13, 20, 64 and 84 among all 86 involved genes based on *t*-test *p*-values. In a comparison with data-only results in Additional file 1: S6.1, 14 different differential edges are found. We also applied a bootstrap method in [37] to assess the robustness of the findings as detailed in Additional file 1: S6.2.

### Apoptosis pathway in patients with early recurrent and non-recurrent breast cancer

Network rewiring analysis is also applicable to mechanistic studies and helps identify underlying key players that cause phenotypic differences. For example, 50% of estrogen receptor-positive breast cancers recur following treatment, but the mechanisms involved in cancer recurrence remain unknown. Understanding of the mechanisms of breast cancer recurrence can provide critical information for early detection and prevention. We used gene expression data from a clinical study [38] to learn differences in the apoptosis pathway in primary tumors between patients with recurrent and non-recurrent disease. We compared the pathway changes in tumors obtained from patients whose breast cancer recurred within 5 years after treatment and from patients who remained recurrence-free for at least 8 years after treatment. There were 47 and 48 tumor samples in the recurring and non-recurring groups, respectively. Gene expression data were generated using Affymetrix U133A arrays. We used the apoptosis pathway from KEGG as prior knowledge.

**Figure 6:**
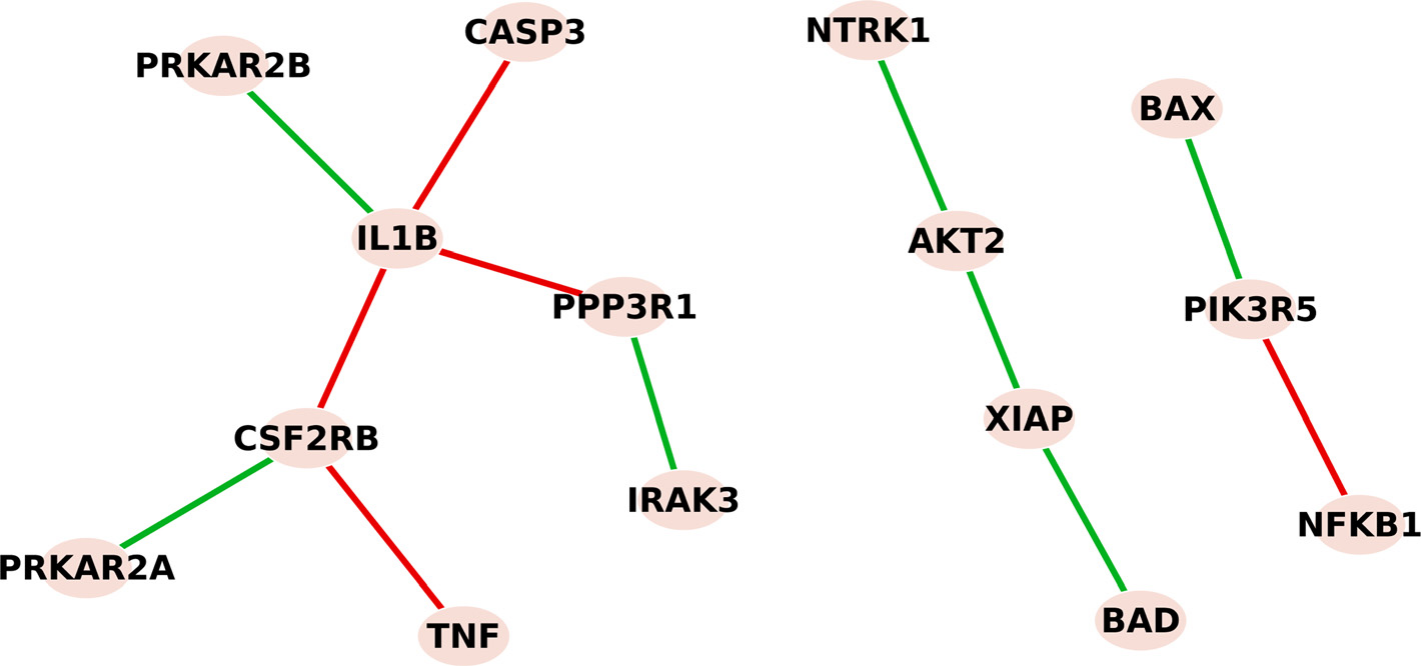
**Rewiring of apoptosis pathway in breast cancer patients with and without recurrence.** Red edges are unique connections in patients with recurrent breast cancer, where inflammatory/immune response genes *IL1B*, *NFκB* and *TNFα* that are all linked to increased resistance to breast cancer treatment were found. Green edges are unique connections in patients with breast cancer that did not recur, where paths to both anti-apoptotic *XIAP*/*AKT2* and proapoptotic *BAX* and *BAD* were formed.

Following the same presentation as in the yeast study, red edges are connections established in patients with recurrent disease, and green edges are connections unique to patients without recurrent disease. Differences in the signaling among genes in the apoptosis pathway between patients whose cancer recurred or who remained cancer-free are shown in Figure 6.

Three inflammatory/immune response genes (*IL1B*, *NFκB* and *TNFα*) that are all linked to increased resistance to breast cancer treatment were identified in the recurrent tumors. These genes formed a path to inhibit proapoptotic *CASP3* and *PPP3R1*[39], and to activate the pro-survival genes *PIK3R5* or *CSF2RB* that maintain cell survival. In contrast, green edges that were present in non-recurrent tumors form paths to both anti-apoptotic *XIAP*/*AKT2* and proapoptotic *BAX* and *BAD* gene functions.

**Figure 7:**
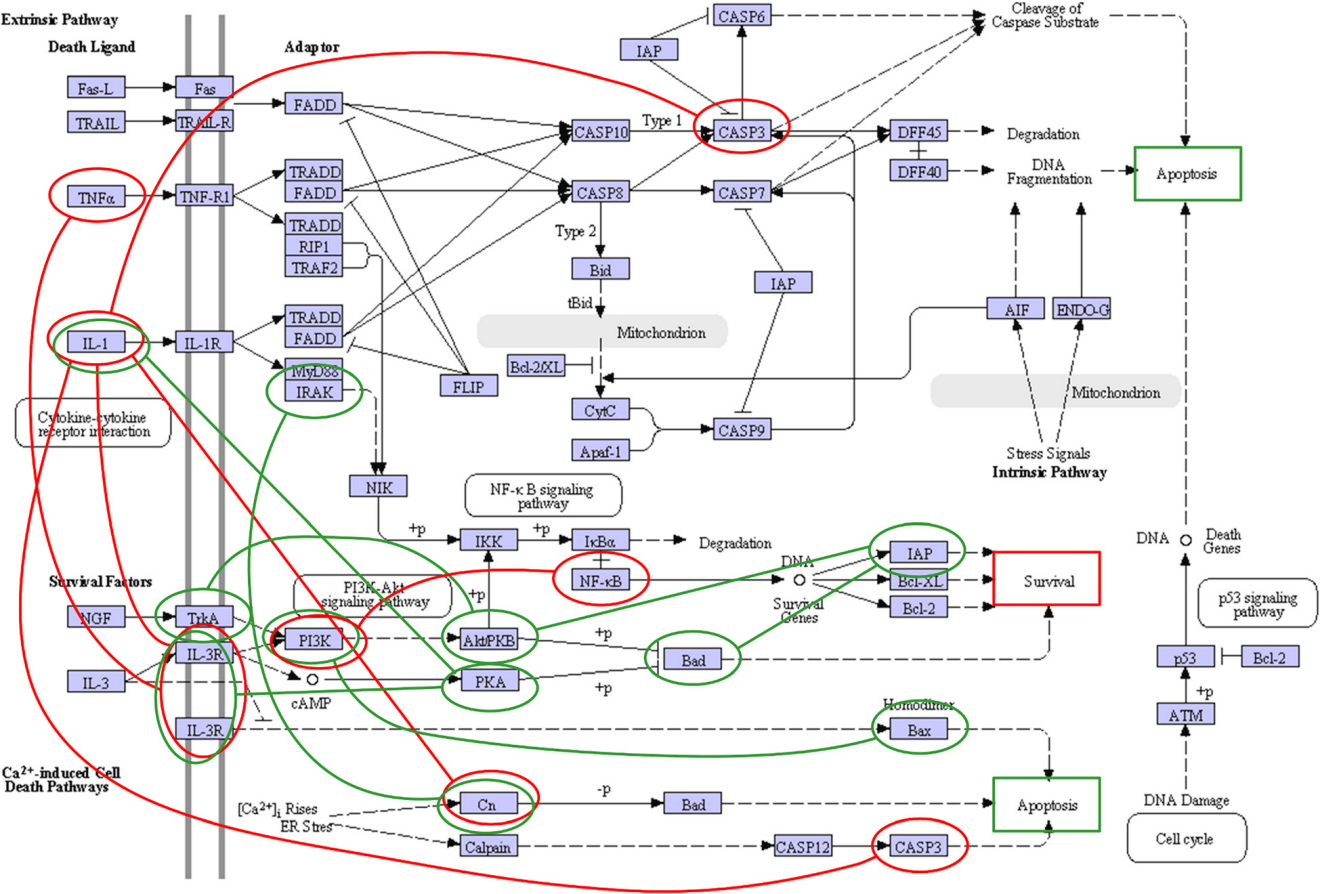
**Differential dependency network presented over the KEGG[**[19]**] apoptosis pathway.** Recurrent breast cancers (featured by red edges) showed the imbalance between apoptosis and survival with only one route into the cell through *IL1B*-induced inhibition of proapoptotic *CASP3*. Non-recurrent breast cancer had a cascade of signaling pathways inside the cell that provides the balance between apoptosis and survival.

When we overlaid the differential network over the KEGG [19] apoptosis pathway we noticed additional differences in the signaling patterns. Using the same color-coded presentation, we show the learned differential network in Figure 7. In the recurrent breast cancers (red edges), the molecular activities mainly affect the initial apoptotic signals outside the cell and within the cell membrane (ligands and their receptors), while inside the cell there is no clear signaling cascade affected to determine cell fate. The only route affected within the cell is *IL1B*-induced inhibition of proapoptotic *CASP3*. In non-recurrent breast cancer, the affected network involves both signals received from activation of the membrane receptors and a cascade of signaling pathways inside the cell to promote both apoptosis and survival. Since a balance between apoptosis and survival is necessary for damaged cells to be eliminated and repaired cells to survive [40], it is logical that both pathways would be activated concurrently. Interestingly, the imbalance of apoptotic and survival signals and the inhibition of *CASP3* in recurrent cancer both lead to the resistance of cell death, reported as a major hallmark of cancer [41].

In conclusion, the apoptosis pathway rewiring analysis identified key mechanistic signaling differences in tumors from patients whose breast cancer did or did not recur. These differences provide a promising ground for novel hypotheses to determine factors affecting breast cancer recurrence.

## Conclusions

To address the challenges concerning differential network inference using data-knowledge integrated approaches, we formulated the problem of learning the condition‐specific network structure and topological changes as a convex optimization problem. Model regularization and prior knowledge were utilized to navigate through the vast solution space. An efficient algorithm was developed to make the solution scalable by exploring the special structure of the problem. Prior knowledge was carefully and efficiently incorporated in seeking the balance between the prior knowledge support and data-derived evidence. The proposed method can efficiently utilize prior knowledge in the network inference while remaining robust to false positive edges in the knowledge. The statistical significance of rewiring and desired type I error rate were assessed and validated. We evaluated the proposed method using synthetic data sets in various cases to demonstrate the effectiveness of this method in learning both common and differential networks, and the simulation results further corroborated our theoretical analysis. We then applied this approach to yeast oxidative stress data to study the cellular dynamic response to this environmental stress by rewiring network structures. Results were highly consistent with previous findings, providing meaningful biological insights into the problem. Finally, we applied the methods to breast cancer recurrence data and obtained biologically plausible results. In the future, we plan to incorporate more types of biological prior information, e.g., protein‐DNA binding information in ChIP‐chip data and protein‐protein interaction data, to improve the use of condition-specific prior knowledge.

## Abbreviations

kDDN: Knowledge-fused differential dependency network

TP: True positives

FP: False positives

kDDN.dt: kDDN with no knowledge

kDDN.tk: kDDN with true knowledge

kDDN.fk: kDDN with random knowledge

## Competing interests

The authors declare that they have no competing interests.

## Authors’ contributions

YT and BZ designed the method and drafted the manuscript along with ZZ and YW. YT and BZ carried out the experimental validation of the methods. EPH, RC, IMS, JX and DMH helped with the methodology development and provided important biological interpretation of the results. RC and DMH helped with the yeast study. RC, ZZ and IMS helped with the breast cancer study. EPH helped with the muscular dystrophy study. All authors read and approved the final manuscript.

## Additional file

## Supplementary Material

Additional file 1: **Supplementary methods and experimental results.** Details of theoretical proofs and algorithms, more synthetic and real data comparisons, and validations are included in this file. Click here for file

## References

[B1] Mitra K Carvunis AR Ramesh SK Ideker T Integrative approaches for finding modular structure in biological networks Nat Rev Genet 2013 14 10 719 732 24045689 10.1038/nrg3552 PMC3940161

[B2] Creixell P Schoof EM Erler JT Linding R Navigating cancer network attractors for tumor-specific therapy Nat Biotechnol 2012 30 9 842 848 22965061 10.1038/nbt.2345

[B3] Califano A Rewiring makes the difference Mol Syst Biol 2011 7 463 21245848 10.1038/msb.2010.117 PMC3049406

[B4] Barabasi AL Gulbahce N Loscalzo J Network medicine: a network-based approach to human disease Nat Rev Genet 2011 12 1 56 68 21164525 10.1038/nrg2918 PMC3140052

[B5] Ideker T, Krogan NJ: **Differential network biology.** *Mol Syst Biol* 2012, **8**(1). 10.1038/msb.2011.99 PMC3296360 22252388

[B6] Reverter A Hudson NJ Nagaraj SH Perez-Enciso M Dalrymple BP Regulatory impact factors: unraveling the transcriptional regulation of complex traits from expression data Bioinformatics 2010 26 7 896 904 20144946 10.1093/bioinformatics/btq051

[B7] Hudson NJ Dalrymple BP Reverter A Beyond differential expression: the quest for causal mutations and effector molecules BMC Genomics 2012 13 356 22849396 10.1186/1471-2164-13-356 PMC3444927

[B8] Shmulevich I Dougherty ER Kim S Zhang W Probabilistic Boolean networks: a rule-based uncertainty model for gene regulatory networks Bioinformatics 2002 18 2 261 274 11847074 10.1093/bioinformatics/18.2.261

[B9] Rangel C Angus J Ghahramani Z Lioumi M Sotheran E Gaiba A Wild DL Falciani F Modeling T-cell activation using gene expression profiling and state-space models Bioinformatics 2004 20 9 1361 1372 14962938 10.1093/bioinformatics/bth093

[B10] Tyson JJ Baumann WT Chen C Verdugo A Tavassoly I Wang Y Weiner LM Clarke R Dynamic modelling of oestrogen signalling and cell fate in breast cancer cells Nat Rev Cancer 2011 11 7 523 532 21677677 10.1038/nrc3081 PMC3294292

[B11] Friedman N Inferring cellular networks using probabilistic graphical models Science 2004 303 5659 799 805 14764868 10.1126/science.1094068

[B12] Hudson NJ Reverter A Dalrymple BP A differential wiring analysis of expression data correctly identifies the gene containing the causal mutation PLoS Comput Biol 2009 5 5 e1000382 19412532 10.1371/journal.pcbi.1000382 PMC2671163

[B13] Zhang B Li H Riggins RB Zhan M Xuan J Zhang Z Hoffman EP Clarke R Wang Y Differential dependency network analysis to identify condition-specific topological changes in biological networks Bioinformatics 2009 25 4 526 532 19112081 10.1093/bioinformatics/btn660 PMC2642641

[B14] Zhang B Tian Y Jin L Li H Shih Ie M Madhavan S Clarke R Hoffman EP Xuan J Hilakivi-Clarke L Wang Y DDN: a caBIG(R) analytical tool for differential network analysis Bioinformatics 2011 27 7 1036 1038 21296752 10.1093/bioinformatics/btr052 PMC3065688

[B15] Roy S Werner-Washburne M Lane T A multiple network learning approach to capture system-wide condition-specific responses Bioinformatics 2011 27 13 1832 1838 21551143 10.1093/bioinformatics/btr270 PMC3117363

[B16] Gill R Datta S Datta S A statistical framework for differential network analysis from microarray data BMC Bioinformatics 2010 11 95 20170493 10.1186/1471-2105-11-95 PMC2838870

[B17] Emmert-Streib F The chronic fatigue syndrome: a comparative pathway analysis J Comput Biol 2007 14 7 961 972 17803373 10.1089/cmb.2007.0041

[B18] Ahmed A Xing EP Recovering time-varying networks of dependencies in social and biological studies Proc Natl Acad Sci 2009 106 29 11878 11883 19570995 10.1073/pnas.0901910106 PMC2704856

[B19] Kanehisa M Goto S KEGG: Kyoto encyclopedia of genes and genomes Nucleic Acids Res 2000 28 1 27 30 10592173 10.1093/nar/28.1.27 PMC102409

[B20] Wang Z Xu W San Lucas FA Liu Y Incorporating prior knowledge into Gene Network Study Bioinformatics 2013 29 20 2633 2640 23956306 10.1093/bioinformatics/btt443 PMC3789546

[B21] Zhang B, Wang Y: **Learning Structural Changes of Gaussian Graphical Models in Controlled Experiments.** In *Uncertainty in Artificial Intelligence (UAI 2010).* 2010.

[B22] Tian Y, Zhang B, Shih I-M, Wang Y: **Knowledge-Guided Differential Dependency Network Learning for Detecting Structural Changes in Biological Networks.** In *ACM International Conference on Bioinformatics and Computational Biology.* 2011:254–263.

[B23] Meinshausen N Bühlmann P High-dimensional graphs and variable selection with the Lasso Ann Stat 2006 34 3 1436 1462

[B24] Bunke H Allermann G Inexact graph matching for structural pattern recognition Pattern Recogn Lett 1983 1 4 245 253

[B25] Ikner A Shiozaki K Yeast signaling pathways in the oxidative stress response Mutat Res Fundam Mol Mech Mutagen 2005 569 1–2 13 27 10.1016/j.mrfmmm.2004.09.006 15603750

[B26] Jamieson DJ Oxidative stress responses of the yeast Saccharomyces cerevisiae Yeast 1998 14 16 1511 1527 9885153 10.1002/(SICI)1097-0061(199812)14:16<1511::AID-YEA356>3.0.CO;2-S

[B27] Kuge S Jones N Nomoto A Regulation of yAP-1 nuclear localization in response to oxidative stress EMBO J 1997 16 7 1710 1720 9130715 10.1093/emboj/16.7.1710 PMC1169774

[B28] Costa VMV Amorim MA Quintanilha A Moradas-Ferreira P Hydrogen peroxide-induced carbonylation of key metabolic enzymes in Saccharomyces cerevisiae: the involvement of the oxidative stress response regulators Yap1 and Skn7 Free Radic Biol Med 2002 33 11 1507 1515 12446208 10.1016/s0891-5849(02)01086-9

[B29] Cherry JM Hong EL Amundsen C Balakrishnan R Binkley G Chan ET Christie KR Costanzo MC Dwight SS Engel SR Fisk DG Hirschman JE Hitz BC Karra K Krieger CJ Miyasato SR Nash RS Park J Skrzypek MS Simison M Weng S Wong ED Saccharomyces Genome Database: the genomics resource of budding yeast Nucleic Acids Res 2012 40 D1 D700 D705 22110037 10.1093/nar/gkr1029 PMC3245034

[B30] Gasch AP Spellman PT Kao CM Carmel-Harel O Eisen MB Storz G Botstein D Brown PO Genomic expression programs in the response of yeast cells to environmental changes Mol Biol Cell 2000 11 12 4241 4257 11102521 10.1091/mbc.11.12.4241 PMC15070

[B31] Causton HC Ren B Koh SS Harbison CT Kanin E Jennings EG Lee TI True HL Lander ES Young RA Remodeling of yeast genome expression in response to environmental changes Mol Biol Cell 2001 12 2 323 337 11179418 10.1091/mbc.12.2.323 PMC30946

[B32] Singh K: *Oxidant-Induced Cell Death Mediated By A Rho Gtpase In Saccharomyces cerevisiae. PhD thesis.*: The Ohio State University, Molecular Genetics Department; 2008.

[B33] Lee ME Singh K Snider J Shenoy A Paumi CM Stagljar I Park H-O The Rho1 GTPase acts together with a vacuolar glutathione S-conjugate transporter to protect yeast cells from oxidative stress Genetics 2011 188 4 859 870 21625004 10.1534/genetics.111.130724 PMC3176091

[B34] Petkova MI Pujol-Carrion N de la Torre-Ruiz MA Signal flow between CWI/TOR and CWI/RAS in budding yeast under conditions of oxidative stress and glucose starvation Commun Integr Biol 2010 3 6 555 557 21331237 10.4161/cib.3.6.12974 PMC3038061

[B35] Grant CM Perrone G Dawes IW Glutathione and catalase provide overlapping defenses for protection against hydrogen peroxide in the Yeast Saccharomyces cerevisiae Biochem Biophys Res Commun 1998 253 3 893 898 9918826 10.1006/bbrc.1998.9864

[B36] Lee J Godon C Lagniel G Spector D Garin J Labarre J Toledano MB Yap1 and Skn7 control Two specialized oxidative stress response regulons in yeast J Biol Chem 1999 274 23 16040 16046 10347154 10.1074/jbc.274.23.16040

[B37] Tripathi S Emmert-Streib F Assessment method for a power analysis to identify differentially expressed pathways PLoS One 2012 7 5 e37510 22629411 10.1371/journal.pone.0037510 PMC3356338

[B38] Loi S Haibe-Kains B Desmedt C Lallemand F Tutt AM Gillet C Ellis P Harris A Bergh J Foekens JA Klijn JG Larsimont D Buyse M Bontempi G Delorenzi M Piccart MJ Sotiriou C Definition of Clinically Distinct Molecular Subtypes in Estrogen Receptor–Positive Breast Carcinomas Through Genomic Grade J Clin Oncol 2007 25 10 1239 1246 17401012 10.1200/JCO.2006.07.1522

[B39] Su Z Xin S Xu L Cheng J Guo J Li L Wei Q The calcineurin B subunit induces TNF-related apoptosis-inducing ligand (TRAIL) expression via CD11b–NF-κB pathway in RAW264.7 macrophages Biochem Biophys Res Commun 2012 417 2 777 783 22197822 10.1016/j.bbrc.2011.12.034

[B40] Murphy K Ranganathan V Farnsworth M Kavallaris M Lock R Bcl-2 inhibits Bax translocation from cytosol to mitochondria during drug-induced apoptosis of human tumor cells Cell Death Differ 2000 7 1 102 111 10713725 10.1038/sj.cdd.4400597

[B41] Hanahan D Weinberg Robert A Hallmarks of cancer: the next generation Cell 2011 144 5 646 674 21376230 10.1016/j.cell.2011.02.013

