# Supplementary material for: Knowledge-fused differential dependency network models for detecting significant rewiring in biological networks
Source: BMC Syst Biol. 2014 Jul 24;8:87. doi: 10.1186/s12918-014-0087-1 (PMC4131167; doi:10.1186/s12918-014-0087-1)
Supplement: Additional file 1: — Supplementary methods and experimental results. Details of theoretical proofs and algorithms, more synthetic and real data comparisons, and validations are included in this file. [file s12918-014-0087-1-S1.pdf]

Additional file 1

Knowledge-fused differential dependency network models for detecting significant rewiring in biological networks

Ye Tian [1,†], Bai Zhang [2,†], Eric R. Hoffman [3], Robert Clarke [4], Zhen Zhang [2], Ie-Ming Shih [2], Jianhua Xuan[1], David M. Herrington[5] and Yue Wang[1,*]

[1]Department of Electrical & Computer Engineering, Virginia Tech, Arlington, VA 22203, USA;

[2]Department of Pathology, Johns Hopkins University, Baltimore, MD 21231, USA; [3]Research Center for Genetic Medicine, Children's National Medical Center, Washington, DC 20010, USA

[4]Lombardi Comprehensive Cancer Center, Georgetown University, Washington, DC 20057, USA;

[5]Department of Internal Medicine, Section on Cardiovascular Medicine, Wake Forest School of Medicine, Winston-Salem, NC 27157, USA;

[†]These authors contributed equally

[*]To whom correspondence should be addressed

Contents

S1 Model Derivation under Gaussian Assumption **3**

S2 Proof of Theorem 1 **4**

S3 Maximum Entropy Distribution of Prior Knowledge **6**

S4 Solutions and Algorithms **7**

S4.1 Model Parameter Selection 7

S4.2 Closed-form Solution to the Sub-problem 8

S4.3 Algorithms 13

S5 Additional Simulation Results **14**

S5.1 Simulation Data Generation 14

S5.2 Performance Evaluation 15

S5.3 Simulation Performance in Noise Cases 20

S5.4 Effects of Nonuniform Random Knowledge 25

S5.5 Effects of False Negatives in Prior Knowledge 29

S5.6 Empirical Type I Error Rate for Simulated Data Sets Under the Null Hypothesis 32

S5.7 Performance Comparison 33

S6 Additional Real Data Results **38**

S6.1 Yeast and Breast Cancer Results with $\theta = 0$ 38

S6.2 Robustness Analysis on Yeast Case Study 39

S6.3 A Case Study on Juvenile Dermatomyositis 40

S6.4 A Case Study on Transcription Factor Estrogen Receptor α Regulation . . 42

S1 Model Derivation under Gaussian Assumption

We assume the gene expression follow Gaussian distribution, and then the network learning problem is mathematically equivalent to a Gaussian graphical model learning problem. In Gaussian graphical models, the structural of the graph is mathematically characterized by the inverse of the covariance matrix, $\mathbf{\Sigma}^{-1}$. The non-zeros elements of $\mathbf{\Sigma}^{-1}$ indicate connections between the corresponding nodes on the graph. Therefore, learning the structure of Gaussian graphical models is in essence to identify the sparse (non-zero) structure of $\mathbf{\Sigma}^{-1}$, which takes care of all orders of dependencies.

ℓ_1-regularization has been successfully applied to learn the sparse (non-zero) structure of $\mathbf{\Sigma}^{-1}$ following one of two main approaches: one approach is to apply ℓ_1-regularization to identify the neighborhood of the nodes in the network, one node at a time, proposed by Meinshausen and Bühlmann (Meinshausen and Bühlmann, 2006); and the other approach is to maximize the penalized log-likelihood, proposed in (Banerjee *et al.*, 2008). There is an established link between the two approaches: the latter approach is the exact maximization of the ℓ_1-penalized log-likelihood, while the former approach, which is also the approach adopted in our paper, is an approximation of the exact problem and asymptotically consistently estimates the set of nonzero elements of $\mathbf{\Sigma}^{-1}$ (Friedman *et al.*, 2008; Meinshausen and Bühlmann, 2006; Banerjee *et al.*, 2008).

While our node by node strategy does make inference faster, the higher order dependencies are still preserved by the method. In fact, we can quickly show that dependencies are captured by this node by node regression theoretically under Gaussian assumption.

Without loss of generalization, we denote p variables follow zero mean multivariate Gaussian distribution:

$$\mathbf{X} = \begin{bmatrix} x_1 \\ x_2 \\ \vdots \\ x_p \end{bmatrix} \sim N(\mathbf{0}, \mathbf{\Sigma}) = C_1 \exp(-\frac{1}{2}\mathbf{X}^T \mathbf{\Sigma}^{-1} \mathbf{X}). \tag{S1}$$

We always move the node being inferred to the position of x_p in $\mathbf{X}$. Let $\mathbf{X}_{-p} =$

$[x_1, x_2, \cdots x_{p-1}]^T$, and

$$\mathbf{\Sigma}^{-1} = \begin{bmatrix} \mathbf{\Sigma}_{11} & \mathbf{\Sigma}_{1p} \\ \mathbf{\Sigma}_{p1} & \Sigma_{pp} \end{bmatrix}^{-1}, \tag{S2}$$

in which $\mathbf{\Sigma}_{11}$ is a $(p-1) \times (p-1)$ matrix.

Using block matrix inverse, we get

$$\begin{aligned}
\mathbf{X}^T\mathbf{\Sigma}^{-1}\mathbf{X} =& \mathbf{X}_{-p}^T(\mathbf{\Sigma}_{11} - \mathbf{\Sigma}_{1p}\Sigma_{pp}^{-1}\mathbf{\Sigma}_{p1})^{-1}\mathbf{X}_{-p} - \mathbf{X}_{-p}^T\mathbf{\Sigma}_{11}^{-1}\mathbf{\Sigma}_{1p}(\Sigma_{pp} - \mathbf{\Sigma}_{p1}\mathbf{\Sigma}_{11}^{-1}\mathbf{\Sigma}_{1p})^{-1}x_p \\
&- x_p\Sigma_{pp}^{-1}\mathbf{\Sigma}_{p1}(\mathbf{\Sigma}_{11} - \mathbf{\Sigma}_{1p}\Sigma_{pp}^{-1}\mathbf{\Sigma}_{p1})^{-1}\mathbf{X}_{-p} + x_p(\Sigma_{pp} - \mathbf{\Sigma}_{p1}\mathbf{\Sigma}_{11}^{-1}\mathbf{\Sigma}_{1p})^{-1}x_p \\
=& \mathbf{X}_{-p}^T\mathbf{\Sigma}_{11}^{-1}\mathbf{X}_{-p} + \mathbf{X}_{-p}^T\mathbf{\Sigma}_{11}^{-1}\mathbf{\Sigma}_{1p}(\Sigma_{pp} - \mathbf{\Sigma}_{p1}\mathbf{\Sigma}_{11}^{-1}\mathbf{\Sigma}_{1p})^{-1}\mathbf{X}_{-p} \\
&- 2\mathbf{X}_{-p}^T\mathbf{\Sigma}_{11}^{-1}\mathbf{\Sigma}_{1p}(\Sigma_{pp} - \mathbf{\Sigma}_{11}^{-1}\mathbf{\Sigma}_{1p})^{-1}x_p + x_p(\Sigma_{pp} - \mathbf{\Sigma}_{p1}\mathbf{\Sigma}_{11}^{-1}\mathbf{\Sigma}_{1p})^{-1}x_p \\
=& \mathbf{X}_{-p}^T\mathbf{\Sigma}_{11}^{-1}\mathbf{X}_{-p} + (x_p - \mathbf{\Sigma}_{p1}\mathbf{\Sigma}_{11}^{-1}\mathbf{X}_{-p})^T(\Sigma_{pp} - \mathbf{\Sigma}_{p1}\mathbf{\Sigma}_{11}^{-1}\mathbf{\Sigma}_{1p})^{-1}(x_p - \mathbf{\Sigma}_{p1}\mathbf{\Sigma}_{11}^{-1}\mathbf{X}_{-p})
\end{aligned} \tag{S3}$$

The conditional probability of x_p

$$\begin{aligned}
P(x_p|\mathbf{X}_{-p}) =& \frac{P(x_p, \mathbf{X}_{-p})}{P(\mathbf{X}_{-p})} \\
=& \frac{C_1 \exp(-\frac{1}{2}\mathbf{X}^T\mathbf{\Sigma}^{-1}\mathbf{X})}{C_2 \exp(-\frac{1}{2}\mathbf{X}_{-p}^T\mathbf{\Sigma}_{11}^{-1}\mathbf{X}_{-p})} \\
=& C_3 \exp(-\frac{1}{2}(x_p - \mathbf{\Sigma}_{p1}\mathbf{\Sigma}_{11}^{-1}\mathbf{X}_{-p})^T(\Sigma_{pp} - \mathbf{\Sigma}_{p1}\mathbf{\Sigma}_{11}^{-1}\mathbf{\Sigma}_{1p})^{-1}(x_p - \mathbf{\Sigma}_{p1}\mathbf{\Sigma}_{11}^{-1}\mathbf{X}_{-p})) \\
\sim& N(x_p - \mathbf{\Sigma}_{p1}\mathbf{\Sigma}_{11}^{-1}\mathbf{X}_{-p}, \Sigma_{pp} - \mathbf{\Sigma}_{p1}\mathbf{\Sigma}_{11}^{-1}\mathbf{\Sigma}_{1p})
\end{aligned} \tag{S4}$$

is also Gaussian distributed. So the maximum likelihood estimation of x_p is

$$x_{p(\mathrm{MLE})} = \mathbf{\Sigma}_{p1}\mathbf{\Sigma}_{11}^{-1}\mathbf{X}_{-p}, \tag{S5}$$

which is boiled down to the linear combination of the remaining nodes.

S2 Proof of Theorem 1

Theorem 1 can be illustrated as Figure S1. Networks are represented by connected clusters of nodes, with gray edges indicating common connections under both conditions, green edges indicating connections uniquely exist under one condition and red edges indicating

connections uniquely exist under the other condition. $G_{\mathbf{T}}$ is the underlying ground-truth network. $G_{\mathbf{X}}$ is the network learned purely from data. δ will control the increase in the error rate induced by random knowledge within the shaded region. By incorporating prior knowledge with good quality, the learning result $G_{\mathbf{X},\mathbf{W}}$ can escape the shaded region and result is significantly improved.

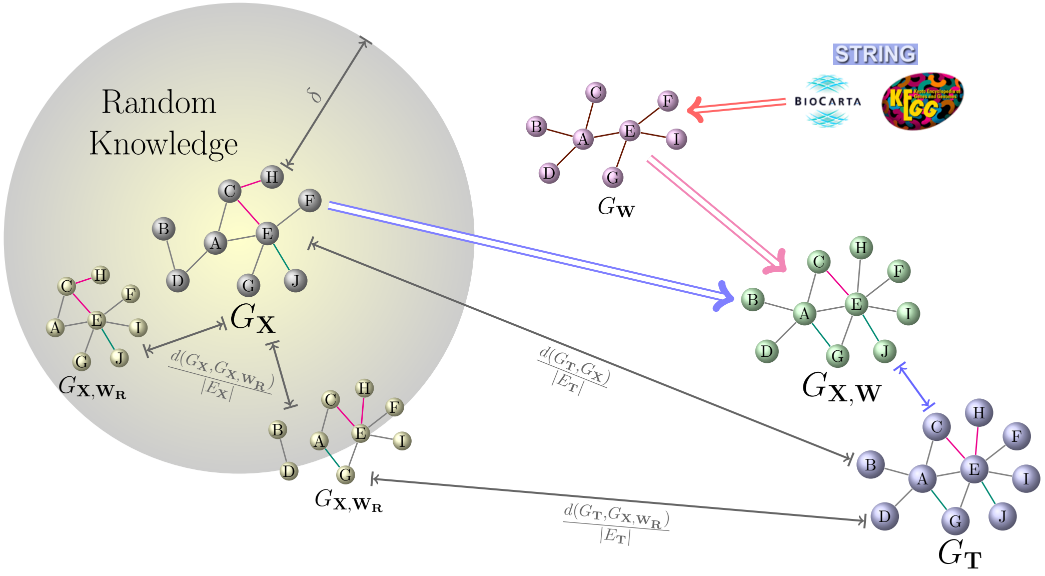

Figure S1: Illustration of theorem 1.

The following is a mathematical proof of the theorem.

Theorem 1. *For a given* $\delta \in [0, 1)$, *if the prior knowledge incorporation parameter* θ *satisfies*

$$\frac{\mathbb{E}[d(G_{\mathbf{X}}, G_{\mathbf{X},\mathbf{W}_{R},\theta})]}{|E_{\mathbf{X}}|} \leq \delta, \tag{S6}$$

then the increase in the error rate induced by incorporating random prior knowledge is bounded by δ, *more specifically,*

$$\frac{\mathbb{E}[d(G_{\mathbf{X},\mathbf{W}_{R},\theta}, G_{\mathbf{T}})]}{|E_{\mathbf{T}}|} \leq \frac{d(G_{\mathbf{T}}, G_{\mathbf{X}})}{|E_{\mathbf{T}}|} + \delta \tag{S7}$$

Proof. The graph edit distance between $G_{\mathbf{X}}$ and $G_{\mathbf{T}}$ is

$$d(G_{\mathbf{T}}, G_{\mathbf{X}}) = FP + FN. \tag{S8}$$

The relationship between $E_{\mathbf{X}}$ and $E_{\mathbf{T}}$ is

$$|E_{\mathbf{X}}| = |E_{\mathbf{T}}| + FP - FN. \tag{S9}$$

The deviation of learned network $G_{\mathbf{X},\mathbf{W}_R,\theta}$ after knowledge incorporation from purely data-driven result $G_{\mathbf{X}}$ is expected to have P wrongly identified edges and N missing edges,

$$\mathbb{E}[d(G_{\mathbf{X}}, G_{\mathbf{X},\mathbf{W}_R,\theta})] = P + N. \tag{S10}$$

Denote the expected number of false positives and false negatives of $G_{\mathbf{X},\mathbf{W}_R,\theta}$ compared to the ground-truth $G_{\mathbf{T}}$ as FP' and FN'. We have

$$FP' \leq FP + P, \tag{S11}$$

$$FN' \leq FN + N. \tag{S12}$$

Therefore,

$$\begin{aligned}
\frac{\mathbb{E}[d(G_{\mathbf{T}}, G_{\mathbf{X},\mathbf{W}_R,\theta})]}{|E_{\mathbf{T}}|} &= \frac{FP' + FN'}{|E_{\mathbf{T}}|} \\
&\leq \frac{FP + FN + P + N}{|E_{\mathbf{T}}|} \\
&= \frac{d(G_{\mathbf{T}}, G_{\mathbf{X}}) + \mathbb{E}[d(G_{\mathbf{X}}, G_{\mathbf{X},\mathbf{W}_R,\theta})]}{|E_{\mathbf{T}}|} \\
&\leq \frac{d(G_{\mathbf{T}}, G_{\mathbf{X}})}{|E_{\mathbf{T}}|} + \delta \frac{|E_{\mathbf{X}}|}{|E_{\mathbf{T}}|} \\
&= \frac{d(G_{\mathbf{T}}, G_{\mathbf{X}})}{|E_{\mathbf{T}}|} + \delta \Big(\frac{|E_{\mathbf{T}}| + FP - FN}{|E_{\mathbf{T}}|} \Big) \\
&\leq \frac{d(G_{\mathbf{T}}, G_{\mathbf{X}})}{|E_{\mathbf{T}}|} + \delta
\end{aligned} \tag{S13}$$

□

S3 Maximum Entropy Distribution of Prior Knowledge

Let $\mathbf{W} \in \mathbb{R}^{p \times p}$ be the (symmetric) adjacency matrix that encodes the "random" prior knowledge. There are M edges in the knowledge and therefore $2M$ elements of $\mathbf{W}$ are "1" and the remaining elements of $\mathbf{W}$ are "0".

Since $\mathbf{W}$ is symmetric, we rearrange the elements of the upper triangle of $\mathbf{W}$ in a vector form, denoted by $\mathbf{x} \in \mathbb{R}^{p(p-1)/2}$. Each element of $\mathbf{x}$ takes values in $\{0, 1\}$. Now we want to find the maximum entropy distribution of $\mathbf{x}$, $P(\mathbf{x})$, given that there are *exactly* M "1" in $\mathbf{x}$. We have

$$\underset{P(\mathbf{x})}{\text{maximize}} \quad H(\mathbf{x}) \tag{S14}$$

$$\text{s.t.} \quad \sum \mathbf{x}[i] = M \tag{S15}$$

The number of possible values taken by $\mathbf{x}$ is $2^{p(p-1)/2}$. However, the number of feasible values that satisfy the equality constraint is $C^M_{p(p-1)/2}$. Denote the set of feasible values of $\mathbf{x}$ as $\mathbb{X}'$. Therefore, the support of the $P(\mathbf{x})$ for the above maximum entropy problem is $\mathbb{X}'$. The entropy of $P(\mathbf{x})$ for the above problem is:

$$H(\mathbf{x}) \tag{S16}$$

$$= -\sum_{\mathbf{x} \in \mathbb{X}'} P(\mathbf{x}) \log P(\mathbf{x}) \tag{S17}$$

Applying Thereom 2.6.4 in (*Elements of Information Theory* by Cover and Thomas), $H(\mathbf{x})$ obtains its maximum when $\mathbf{x}$ is uniformly distributed over $\mathbb{X}'$.

Therefore the maximum entropy distribution for the "random" knowledge is

$$P(\mathbf{x}) = \begin{cases} \frac{1}{C^M_{p(p-1)/2}}, & \mathbf{x} \in \mathbb{X}', \\ 0, & \text{otherwise.} \end{cases} \tag{S18}$$

S4 Solutions and Algorithms

S4.1 Model Parameter Selection

In problem formulation (4), the first ℓ_1-regularization term, $\lambda_1 \sum_{j=1}^{p}(1 - W_{ji}\theta)(|\beta^{(1)}_{ji}| + |\beta^{(2)}_{ji}|)$, leads to the identification of sparse graph structures. Let $\mathbf{W} = \mathbf{0}$ or $\theta = 0$ to examine the performance based solely on data, which regresses to the problem in (Zhang and Wang, 2010), λ_1 is expected to yield a network neither too sparse nor too dense. One

approach to determine λ_1 under Gaussian assumption and $\lambda_2 = 0$ is by controlling the risk of falsely extending connectivity to distinct components in the graph no larger than α_1 (which is typically set to 0.05),

$$\lambda_1 = \frac{2}{N}\left(1 - \Phi(\frac{\alpha_1}{2p^2})\right), \tag{S19}$$

which is outlined by Theorem 3 in (Meinshausen and Bühlmann, 2006).

The second $\ell_1$regularization term, $\lambda_2\|\boldsymbol{\beta}_i^{(1)} - \boldsymbol{\beta}_i^{(2)}\|_1$, works specifically on differential edges to suppress inconsistencies of the network structures and parameters between two conditions. At a given significance level α_2, (e.g., $\alpha_2 = 0.05$ is used in this paper), only differential edges that are statistically significant are expected to enter the differential dependency network. So the λ_2 corresponding to α_2 is found by putting the type I error rate under null distribution in the vicinity of α_2 using batches of permuted samples. Suppose the size of the network or the number of edges in the network is E under null distribution, the expected type I error rate should be α_2, *i.e.*, there are still $\alpha_2 E$ edges falsely claimed as differential edges. λ_2 is then found by gradually increasing its value from 0 until the type I error rate falls into the vicinity of α_2 under null distribution to guarantee desired detection power. The p-value of differential edges can be further assessed using permutation test.

S4.2 Closed-form Solution to the Sub-problem

For notational simplicity, we can always normalize the variables to mean 0 and unit length by location and scale transformations,

$$\begin{aligned} \sum_{k=1}^{N} x_{ki}^{(1)} &= 0, \qquad \sum_{k=1}^{N} (x_{ki}^{(1)})^2 = 1, \\ \sum_{k=1}^{N} x_{ki}^{(2)} &= 0, \qquad \sum_{k=1}^{N} (x_{ki}^{(2)})^2 = 1, \end{aligned} \tag{S20}$$

where $i = 1, 2, \cdots, p$. Here we assume this normalization step has already been performed. Additionally, the orthogonality between j^{th} column of matrix $\mathbf{X}$, $\boldsymbol{x}_j$ and the $(j+p)^{\text{th}}$ column of $\mathbf{X}$, $\boldsymbol{x}_{j+p}$ simplifies the derivation of closed-form solutions to the sub-problems in each iterations of the block coordinate descent.

Since $\beta_{li}^{(1)}$ and $\beta_{li}^{(2)}$, $l = 1, 2, \cdots, p, l \neq j$, are fixed during iteration $r+1$, we rewrite the objective function of (3) as

$$\begin{aligned}
&\tilde{f}(\boldsymbol{\beta}_i) \\
=&\frac{1}{2}\|\boldsymbol{y}_i - \sum_{l\neq i,j}\boldsymbol{x}_l\beta_{li}^{(1),r} - \sum_{l\neq i,j}\boldsymbol{x}_{p+l}\beta_{li}^{(2),r} \\
&\qquad\qquad - \boldsymbol{x}_j\beta_{ji}^{(1)} - \boldsymbol{x}_{p+j}\beta_{ji}^{(2)}\|_2^2 \\
&+ \lambda_1\sum_{l\neq i,j}(1 - W_{li}\theta)(|\beta_{li}^{(1),r}| + |\beta_{li}^{(2),r}|) \\
&+ \lambda_2\sum_{l\neq i,j}(|\beta_{li}^{(1),r} - \beta_{li}^{(2),r}|) \\
&+ \lambda_1(1 - W_{ji}\theta)(|\beta_{ji}^{(1)}| + |\beta_{ji}^{(2)}|) + \lambda_2|\beta_{ji}^{(1)} - \beta_{ji}^{(2)}|
\end{aligned} \tag{S21}$$

Let

$$\tilde{\boldsymbol{y}}_i = \boldsymbol{y}_i - \sum_{l\neq i,j}\boldsymbol{x}_l\beta_{li}^{(1),r} - \sum_{l\neq i,j}\boldsymbol{x}_{p+l}\beta_{li}^{(2),r} \tag{S22}$$

Therefore, updating $(\beta_{ji}^{(1)}, \beta_{ji}^{(2)})$ is equivalent to

$$\begin{aligned}
&(\beta_{ji}^{(1),r+1}, \beta_{ji}^{(2),r+1}) \\
=&\arg\min_{\beta_{ji}^{(1)},\beta_{ji}^{(2)}} \tilde{f}(\boldsymbol{\beta}_i) \\
=&\arg\min_{\beta_{ji}^{(1)},\beta_{ji}^{(2)}} \frac{1}{2}\|\tilde{\boldsymbol{y}}_i - \boldsymbol{x}_j\beta_{ji}^{(1)} - \boldsymbol{x}_{p+j}\beta_{ji}^{(2)}\|_2^2 \\
&\quad + \lambda_1(1 - W_{ji}\theta)(|\beta_{ji}^{(1)}| + |\beta_{ji}^{(2)}|) + \lambda_2|\beta_{ji}^{(1)} - \beta_{ji}^{(2)}|
\end{aligned} \tag{S23}$$

Denote

$$\rho_1 = \tilde{\boldsymbol{y}}_i{}^T \cdot \boldsymbol{x}_j, \tag{S24}$$

$$\rho_2 = \tilde{\boldsymbol{y}}_i{}^T \cdot \boldsymbol{x}_{p+j}. \tag{S25}$$

First, we examine a simple case, the solution, $(\beta_{ji}^{(1)}, \beta_{ji}^{(2)})$, satisfies

$$\begin{cases} \beta_{ji}^{(1)} > 0, \\ \beta_{ji}^{(2)} > 0, \\ \beta_{ji}^{(1)} < \beta_{ji}^{(2)}. \end{cases} \tag{S26}$$

Take derivative of objective function (S23), and we have

$$\begin{aligned} \frac{\partial \tilde{f}}{\partial \beta_{ji}^{(1)}} = \beta_{ji}^{(1)} - \rho_1 + \lambda_1(1 - W_{ji}\theta)\text{sgn}(\beta_{ji}^{(1)}) \\ + \lambda_2 \text{sgn}(\beta_{ji}^{(1)} - \beta_{ji}^{(2)}), \end{aligned} \tag{S27}$$

$$\begin{aligned} \frac{\partial \tilde{f}}{\partial \beta_{ji}^{(2)}} = \beta_{ji}^{(2)} - \rho_2 + \lambda_1(1 - W_{ji}\theta)\text{sgn}(\beta_{ji}^{(2)}) \\ - \lambda_2 \text{sgn}(\beta_{ji}^{(1)} - \beta_{ji}^{(2)}), \end{aligned} \tag{S28}$$

where $\text{sgn}(\cdot)$ is the sign function.

When $\rho_1 > \lambda_1(1 - W_{ji}\theta) - \lambda_2$ and $\rho_2 > \rho_1 + 2\lambda_2$, we have

$$\begin{cases} \beta_{ji}^{(1)} = \rho_1 - \lambda_1(1 - W_{ji}\theta) + \lambda_2, \\ \beta_{ji}^{(2)} = \rho_2 - \lambda_1(1 - W_{ji}\theta) - \lambda_2. \end{cases} \tag{S29}$$

Similarly, we derive all closed-form solutions to problem (S21), depending on the values of ρ_1, ρ_2 with respect to $\lambda_1(1 - W_{ji}\theta), \lambda_2$. The plane (ρ_1, ρ_2) is divided into 13 regions, as shown in Figure S2.

Depending on the location of (ρ_1, ρ_2) in the plane, the solutions to problem (S21) are as follows.

If (ρ_1, ρ_2) is in region (0), then

$$\beta_{ji}^{(1)} = \beta_{ji}^{(2)} = 0. \tag{S30}$$

If (ρ_1, ρ_2) is in region (1), then

$$\beta_{ji}^{(1)} = \beta_{ji}^{(2)} = \frac{1}{2}(\rho_1 + \rho_2) - \lambda_1(1 - W_{ji}\theta) \tag{S31}$$

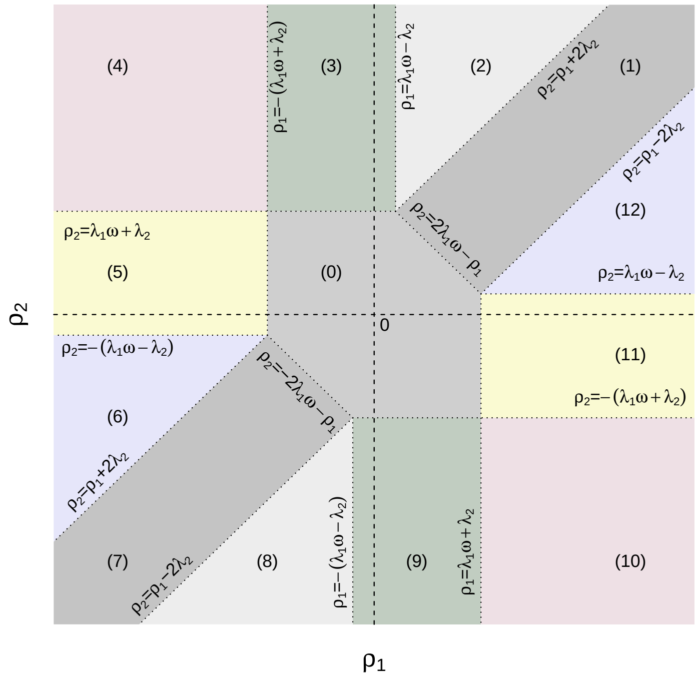

Figure S2: Solution regions of the sub-problem (S21), $\omega = 1 - W_{ji}\theta$.

If (ρ_1, ρ_2) is in region (2), then

$$\begin{cases} \beta_{ji}^{(1)} = \rho_1 - \lambda_1(1 - W_{ji}\theta) + \lambda_2, \\ \beta_{ji}^{(2)} = \rho_2 - \lambda_1(1 - W_{ji}\theta) - \lambda_2. \end{cases} \tag{S32}$$

If (ρ_1, ρ_2) is in region (3), then

$$\begin{cases} \beta_{ji}^{(1)} = 0, \\ \beta_{ji}^{(2)} = \rho_2 - \lambda_1(1 - W_{ji}\theta) - \lambda_2. \end{cases} \tag{S33}$$

If (ρ_1, ρ_2) is in region (4), then

$$\begin{cases} \beta_{ji}^{(1)} = \rho_1 + \lambda_1(1 - W_{ji}\theta) + \lambda_2, \\ \beta_{ji}^{(2)} = \rho_2 - \lambda_1(1 - W_{ji}\theta) - \lambda_2. \end{cases} \tag{S34}$$

If (ρ_1, ρ_2) is in region (5), then

$$\begin{cases} \beta_{ji}^{(1)} = \rho_1 + \lambda_1(1 - W_{ji}\theta) + \lambda_2, \\ \beta_{ji}^{(2)} = 0. \end{cases} \tag{S35}$$

If (ρ_1, ρ_2) is in region (6), then

$$\begin{cases} \beta_{ji}^{(1)} = \rho_1 + \lambda_1(1 - W_{ji}\theta) + \lambda_2, \\ \beta_{ji}^{(2)} = \rho_2 + \lambda_1(1 - W_{ji}\theta) - \lambda_2. \end{cases} \tag{S36}$$

If (ρ_1, ρ_2) in region (7), then

$$\beta_{ji}^{(1)} = \beta_{ji}^{(2)} = \frac{1}{2}(\rho_1 + \rho_2) + \lambda_1(1 - W_{ji}\theta). \tag{S37}$$

If (ρ_1, ρ_2) is in region (8), then

$$\begin{cases} \beta_{ji}^{(1)} = \rho_1 + \lambda_1(1 - W_{ji}\theta) - \lambda_2, \\ \beta_{ji}^{(2)} = \rho_2 + \lambda_1(1 - W_{ji}\theta) + \lambda_2. \end{cases} \tag{S38}$$

If (ρ_1, ρ_2) is in region (9), then

$$\begin{cases} \beta_{ji}^{(1)} = 0, \\ \beta_{ji}^{(2)} = \rho_2 + \lambda_1(1 - W_{ji}\theta) + \lambda_2. \end{cases} \tag{S39}$$

If (ρ_1, ρ_2) is in region (10), then

$$\begin{cases} \beta_{ji}^{(1)} = \rho_1 - \lambda_1(1 - W_{ji}\theta) - \lambda_2 \\ \beta_{ji}^{(2)} = \rho_2 + \lambda_1(1 - W_{ji}\theta) + \lambda_2. \end{cases} \tag{S40}$$

If (ρ_1, ρ_2) is in region (11), then

$$\begin{cases} \beta_{ji}^{(1)} = \rho_1 - \lambda_1(1 - W_{ji}\theta) - \lambda_2 \\ \beta_{ji}^{(2)} = 0. \end{cases} \tag{S41}$$

If (ρ_1, ρ_2) is in region (12), then

$$\begin{cases} \beta_{ji}^{(1)} = \rho_1 - \lambda_1(1 - W_{ji}\theta) - \lambda_2 \\ \beta_{ji}^{(2)} = \rho_2 - \lambda_1(1 - W_{ji}\theta) + \lambda_2. \end{cases} \tag{S42}$$

S4.3 Algorithms

Algorithm S1 uses block coordinate decent to solve the node-wise problem and is embedded in Algorithm S2 to determine the optimal degree of knowledge incorporation.

Algorithm S1 Block coordinate descent algorithm to solve problem (4)

Initialization: $\boldsymbol{\beta}_i^0 = [0, 0, ..., 0]$, $r = 0$

while $\boldsymbol{\beta}_i^r$ is not converged **do**

  $j \leftarrow (r \mod p) + 1$

  if $j \neq i$ **then**

    Let $\beta_{li}^{(1),r+1} = \beta_{li}^{(1),r}$, $\beta_{li}^{(2),r+1} = \beta_{li}^{(2),r}$, $l \neq j$

    Solve the jth sub-problem using (S22), (S24), (S25) and (S30)-(S42) in section S4.2.

  end if

  $r \leftarrow r + 1$

end while

Algorithm S2 Sampling and estimation method to solve problem (6)

Inputs: HIGH=0.9, LOW=0.1, MID=(HIGH-LOW)/2+LOW, $B(=1000), M$

Initialization: θ =MID, $D = 0$

Solve problem (4) with $\mathbf{W} = \mathbf{0}$ using Algorithm S1, get $G_{\mathbf{X}}$, $|E_{\mathbf{X}}|$

while HIGH-LOW< 0.01 **do**

  for $i = 1$ **to** B **do**

    Let $\mathbf{W}_R = \mathbf{0}$

    Sample M elements in the upper triangle of $\mathbf{W}_R$, $(a_j, b_j), j = 1, 2, \cdots, M$

    Let $\mathbf{W}_R(a_j, b_j) = 1, \mathbf{W}_R(b_j, a_j) = 1$

    Solve problem (4) with $\mathbf{W}_R$ using Algorithm S1, get $G_{\mathbf{W}_R}$

    Calculate $d_i = d(G_{\mathbf{X}}, G_{\mathbf{X},\mathbf{W}_R,\theta})$

    Let $D = D + d_i$

  end for

  if $\frac{D}{B|E_{\mathbf{X}}|} > \delta$ **then**

    HIGH=MID

  else

    LOW=MID

  end if

  MID=(HIGH-LOW)/2+LOW

end while

Output: θ =MID

S5 Additional Simulation Results

S5.1 Simulation Data Generation

In the simulation studies, we used Gaussian Markov random field to generate the simulation data following four steps. Firstly, generate two adjacency matrices with sparse changes. Secondly, create the precision matrix with same structures with the adjacency matrices.

Thirdly, get valid covariance matrices by inverting the precision matrices. Lastly, simulate data according to the covariance matrices.

A network structure can be represented by an adjacency matrix, where non-zeros indicate dependencies between nodes. We used Gaussian Markov random field to generate the simulation data. Under such model, nodes follow multivariate Gaussian distribution and their dependencies are reflected by the non-zero elements in precision matrix, which can be equivalently treated as the adjacency matrix of a network. So we generate an $N \times N$ precision matrix, then the network structure and simulation data can be derived from the precision matrix. In the generation of precision matrix, we first initialize an $N \times N$ empty matrix, and then every node is randomly connected to d neighbors. d is the degree of connection uniformly chosen between 1 and 4 to get a sparse structure. Nodes with more than d connections are performed with random connection removal and nodes with no connections are performed with random connection addition until all nodes have connections but no more than 4. In order to make the precision matrix invertible and invert to a valid positive semi-definite covariance matrix, we randomly assign and adjust the values in precision matrix in the range [0.2, 0.3], while keeping the sum of each row less than 1 (Meinshausen and Bühlmann, 2006). The simulation data is finally generated according to covariance matrix using R package mvtnorm (Genz *et al.*, 2012; Genz and Bretz, 2009).

S5.2 Performance Evaluation

To evaluate the network learning performance in precision and recall, we created 100 replicate networks, each with 150 samples. For each network, precision and recall of purely data-driven kDDN, naïve baseline and kDDN with prior knowledge are calculated as the false positive rate of prior knowledge gradually increases. The results of all 100 replicates are averaged. The mean and one standard deviation are plotted in Figures S3-S6. The results show that prior knowledge incorporation has the same designed effect irrespective to particular simulation realization.

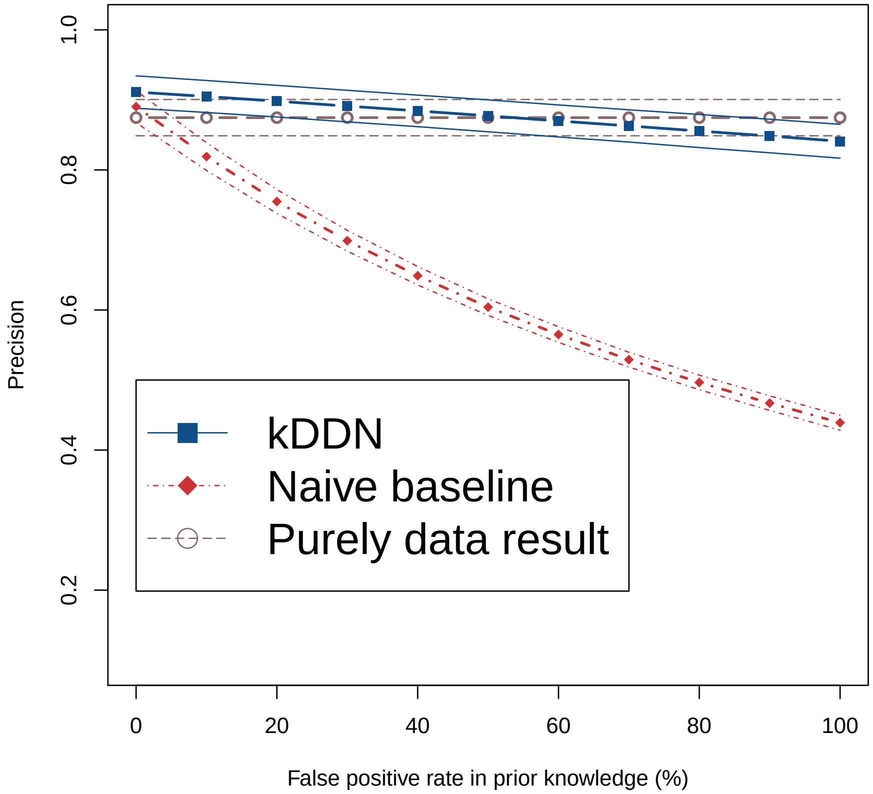

Figure S3: Average precision of overall network learning.

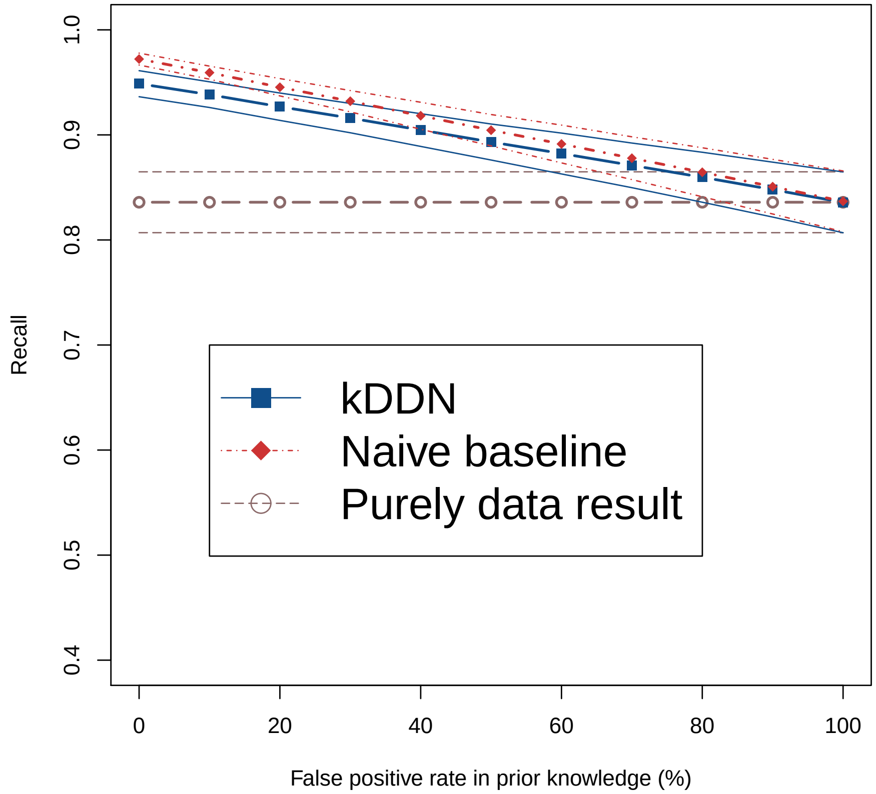

Figure S4: Average recall of overall network learning.

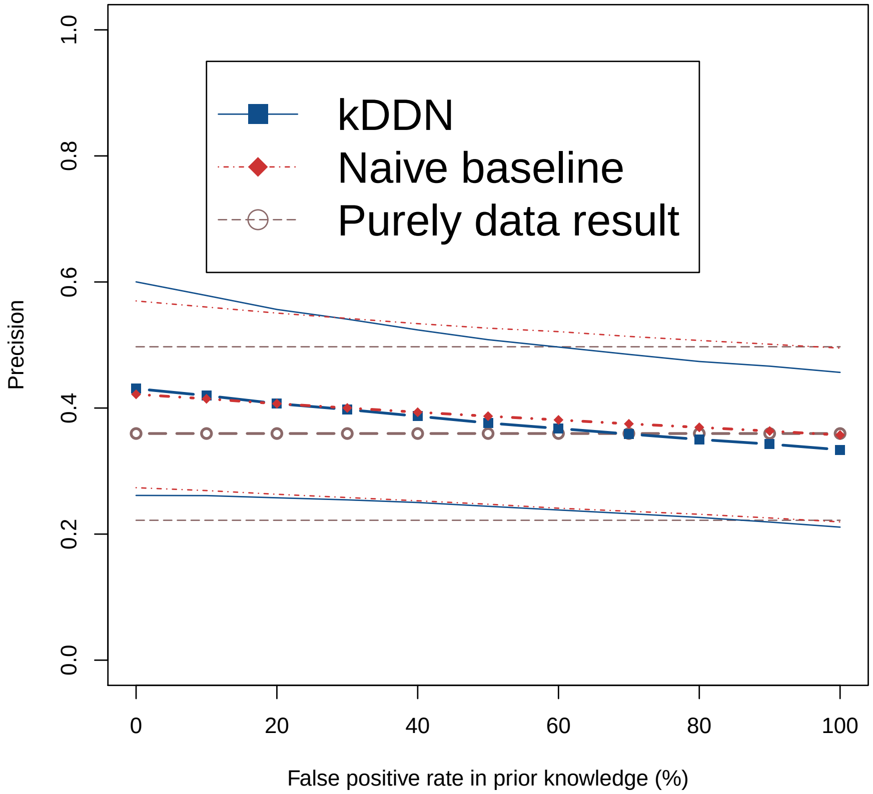

Figure S5: Average precision of differential network learning.

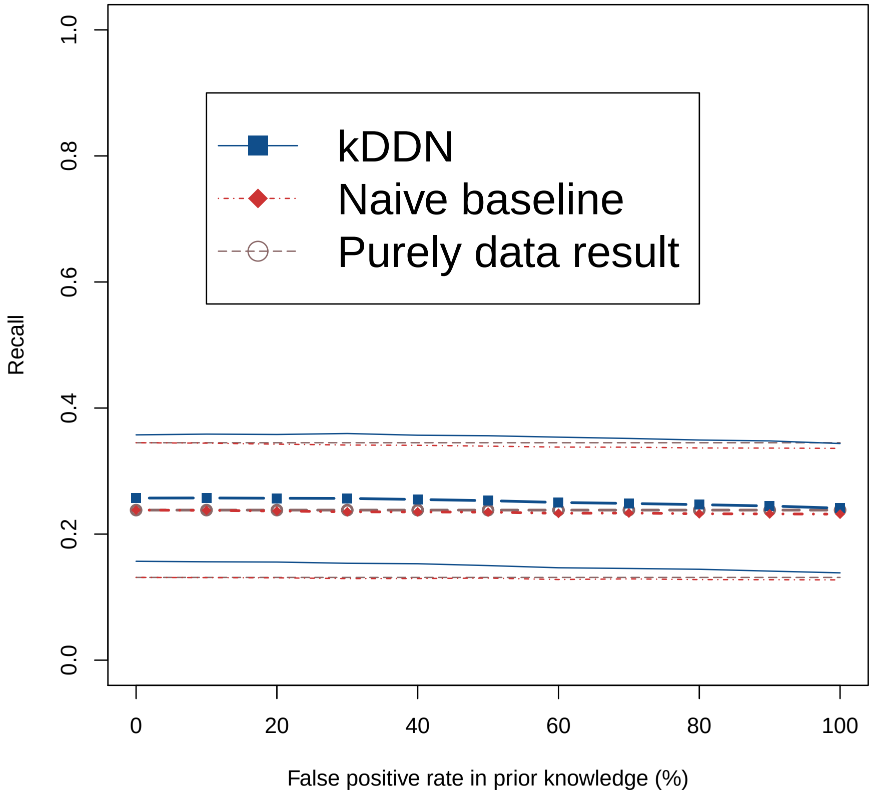

Figure S6: Average recall of differential network learning.

S5.3 Simulation Performance in Noise Cases

We demonstrate the performance of the methods with noise corrupted simulation data. We fix the sample size at 100, and generated simulation data for $p = 50, 100, 200$ added Gaussian white noise with signal to noise ratio $SNR = 0, 1, 2, 3, 4, 5$. For each case we compare the performance of the method under: purely data without noise, purely data with noise, and gradually increase false positives in knowledge with and without noise.

The results of data sets with $p = 50$ are in Figure S7.

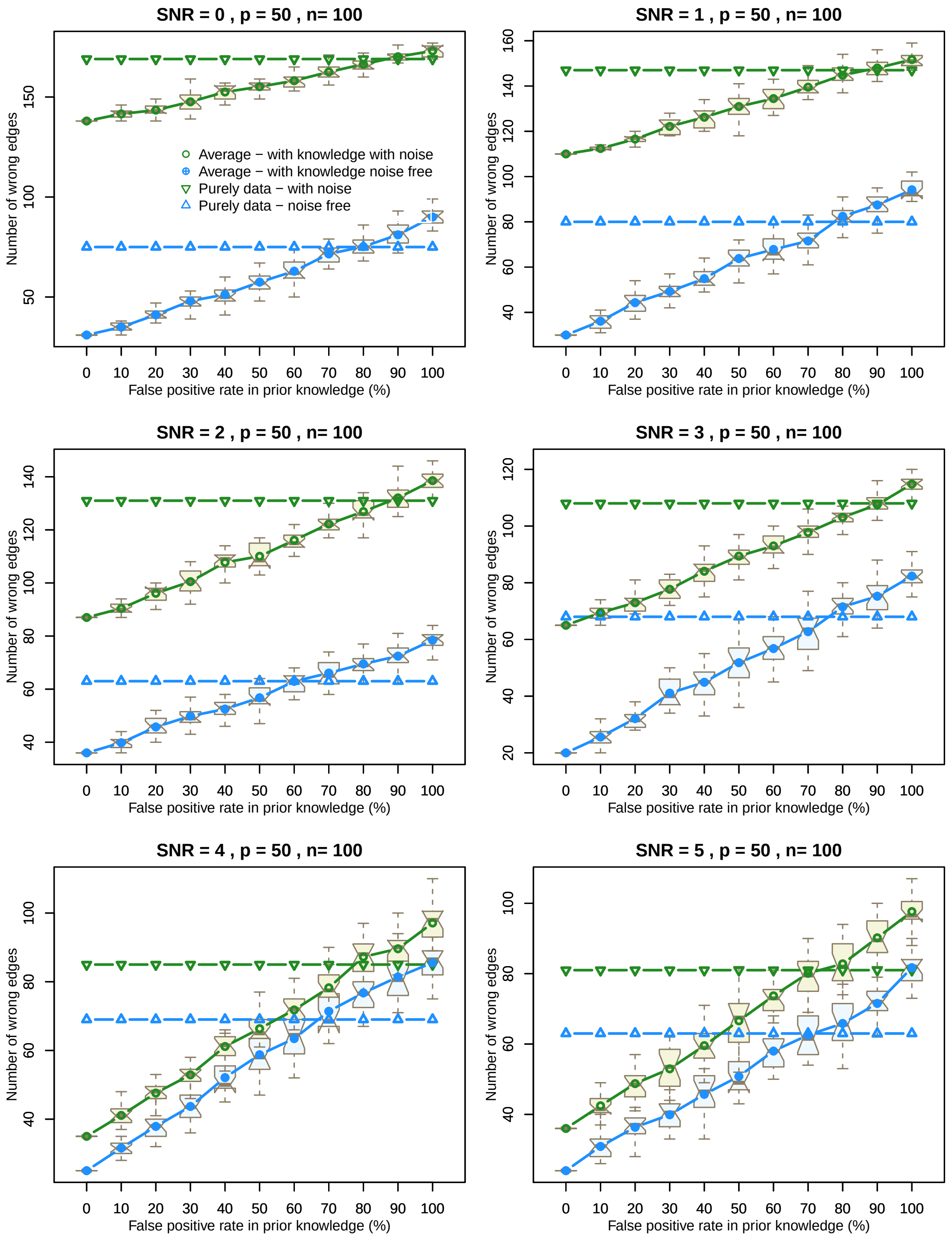

Figure S7: Performance in noise cases, p=50.

From the results we still see the effectiveness of knowledge incorporation in all noise level. On one hand, the performance degrades as a result of noise corruption compared with noise free. But on the other hand we are happy to see that in all noise level knowledge incorporation largely improved the performance and the adverse effects of all wrong malicious knowledge.

The experiment results with $p = 100$ and $p = 200$ are in Figures S8 and S9, from which we can see the similar trends and conclusions.

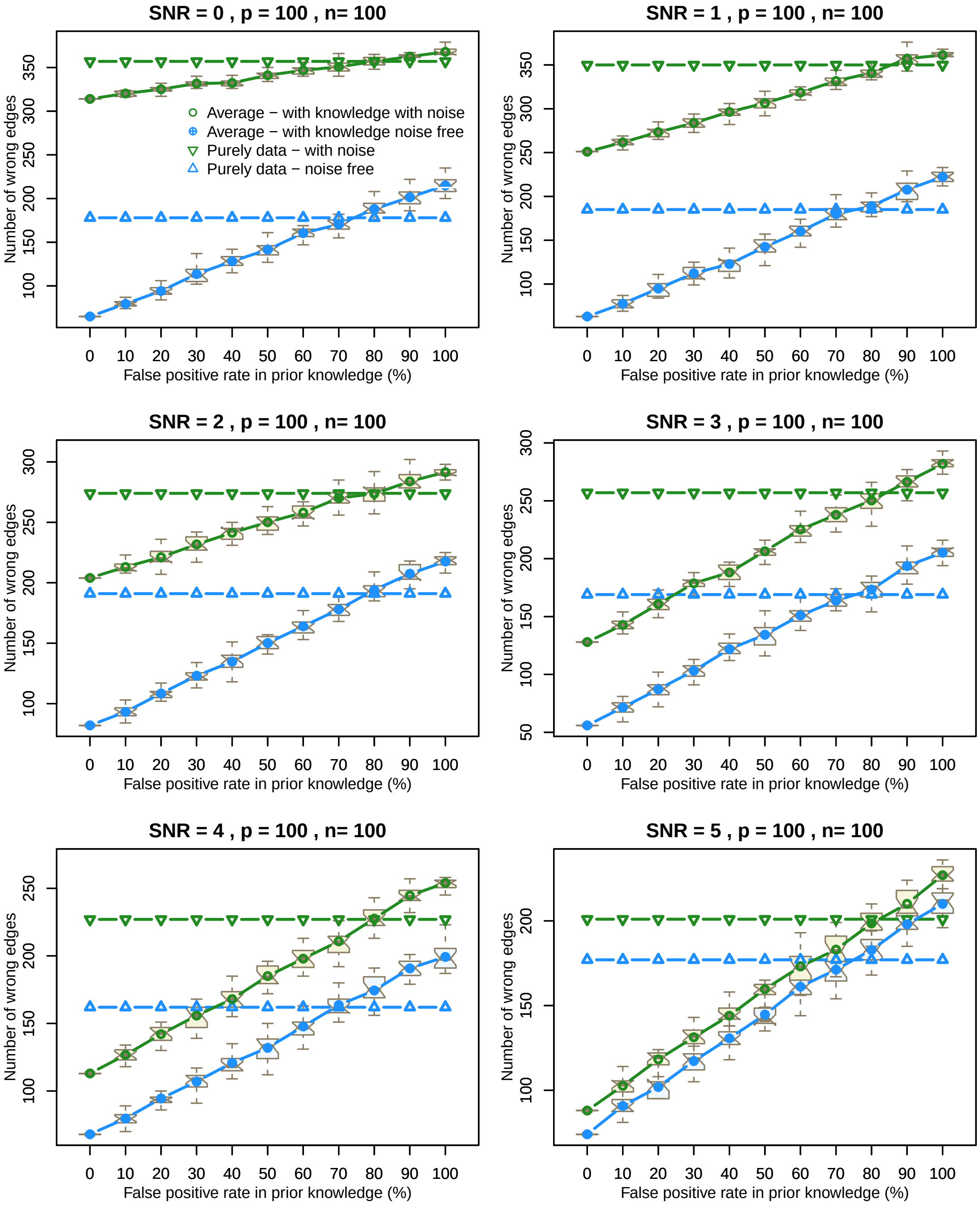

Figure S8: Performance in noise cases, p=100.

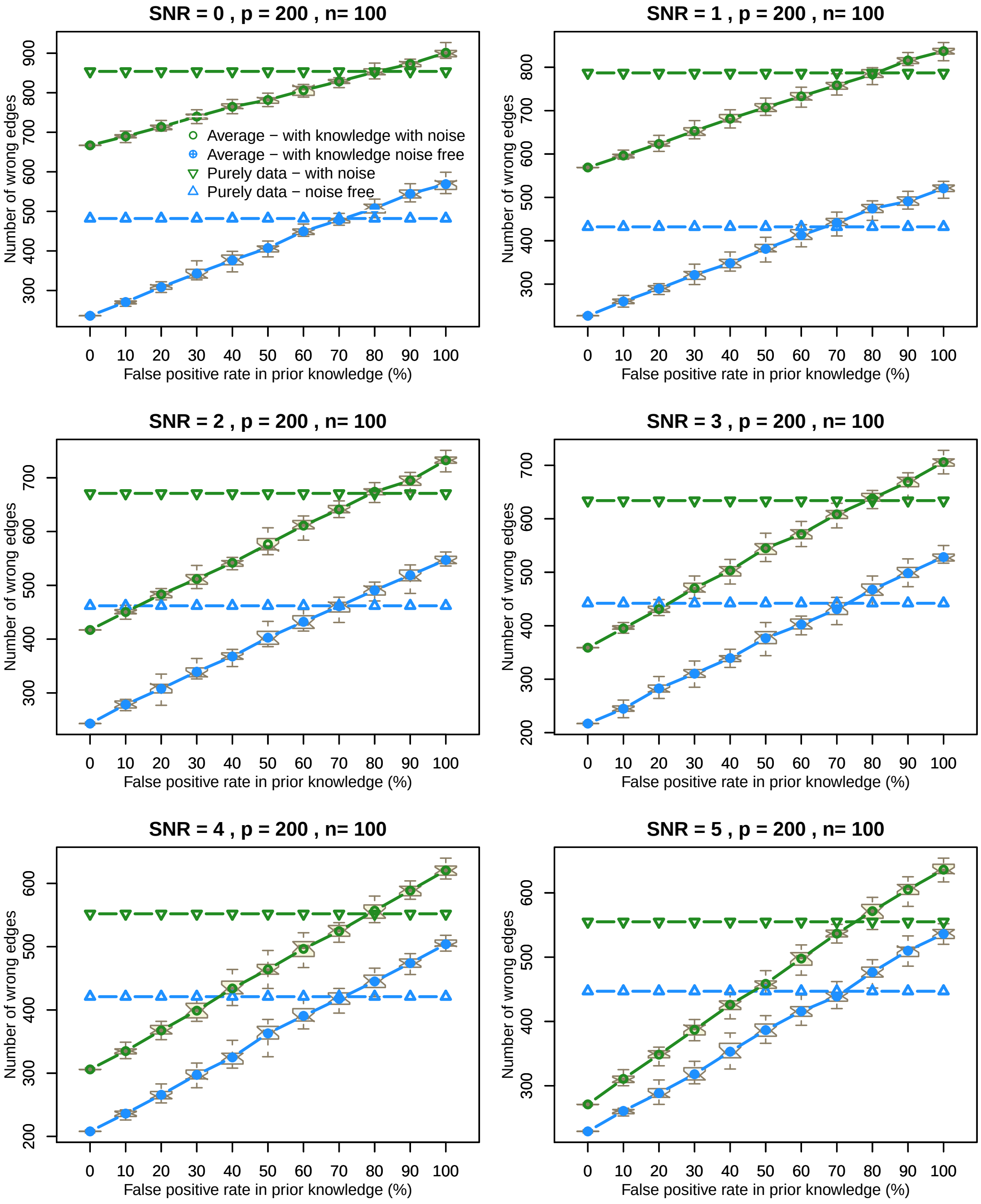

Figure S9: Performance in noise cases, p=200.

S5.4 Effects of Nonuniform Random Knowledge

In practice, FPs in prior knowledge may be more likely to bias towards or against certain nodes rather than distributed uniformly. Proteins/genes with important functions tend to be studied more intensively and therefore accumulated more knowledge. Under specific conditions, knowledge associated with those proteins/genes are more likely to include FPs. In such cases, FPs concentrate more on some nodes than others, which actually makes kDDN avoid FPs more efficiently due to sparse selection mechanism. We compared the performance with random knowledge and biased knowledge using simulation data. Instead of adding false positives uniformly, we add false positives to nodes as follows to biased towards top nodes: the first node contains 1/3 of all FPs, the second node contains 1/3 of the remaining FPs which is 2/9, and the third node contains 1/3 of the remaining FPs, etc. Since in practice the knowledge and data are independent and the data generation process is equal for all nodes, the order of nodes does not matter in this biased knowledge assignment. The results are shown by Figures S10-S13 for 100 node example. This change of random knowledge generation either made no difference or slightly improved the performance, confirming that the uniformly random is the worst case and kDDN bounds the performance under worst case scenario.

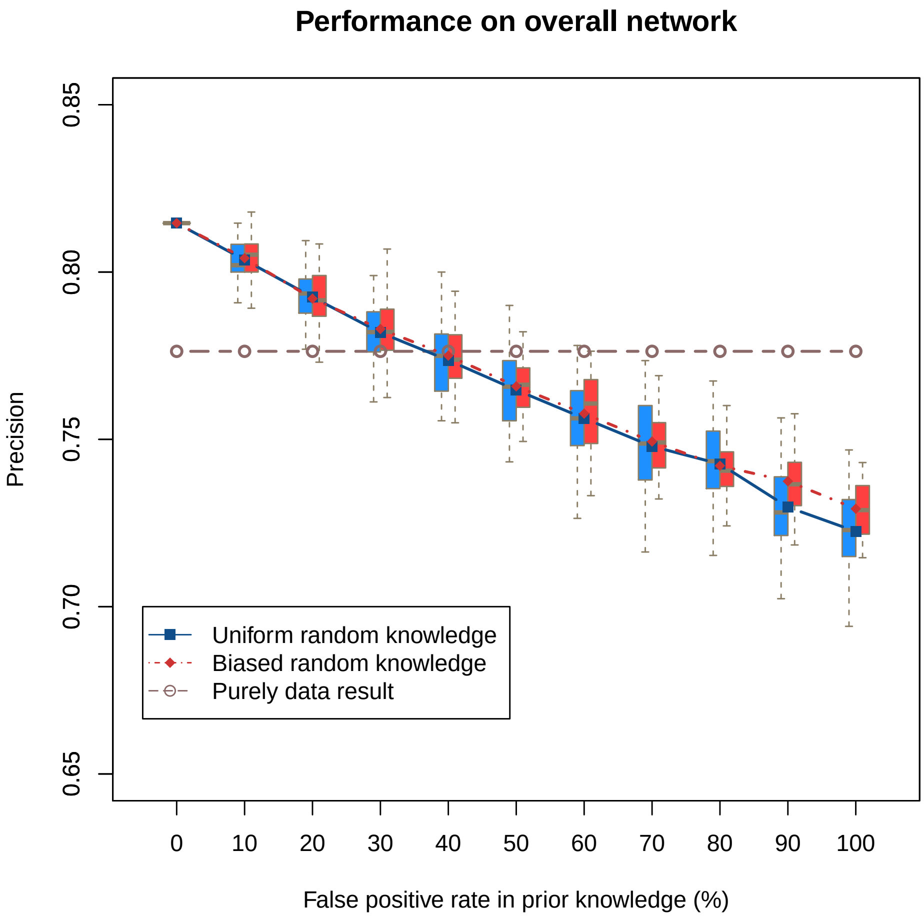

Figure S10: The effects of nonuniform random prior knowledge on inference precision of overall network.

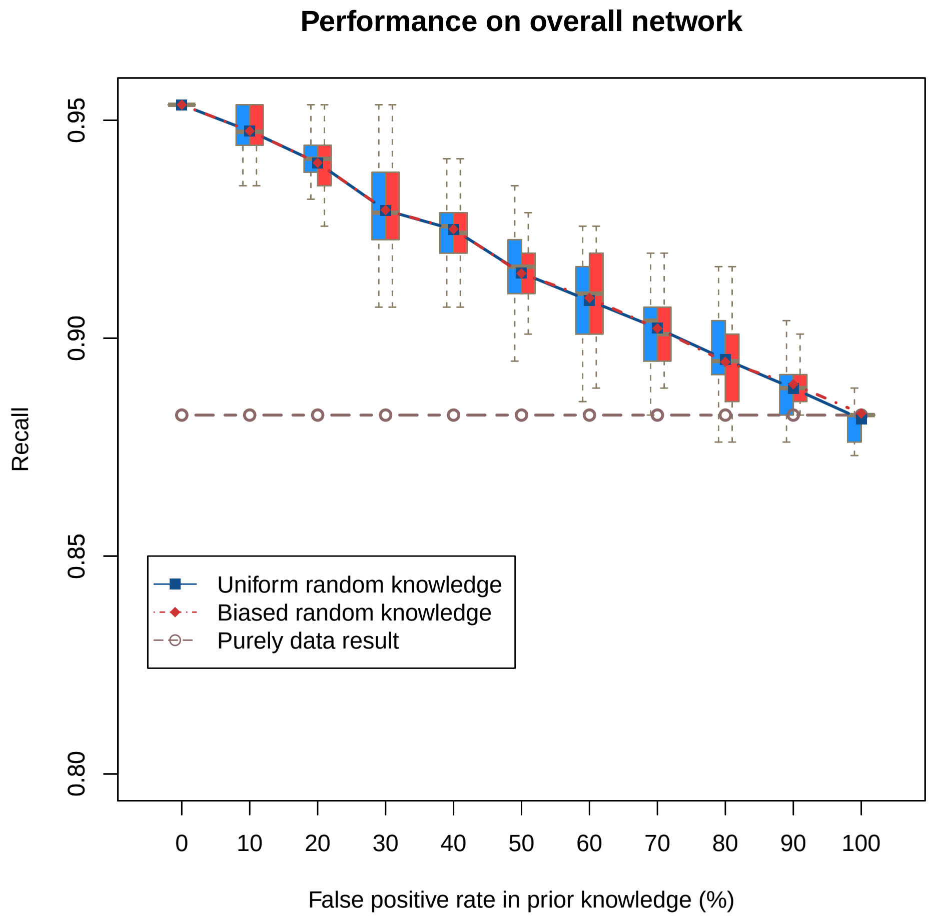

Figure S11: The effects of nonuniform random prior knowledge on inference recall of overall network.

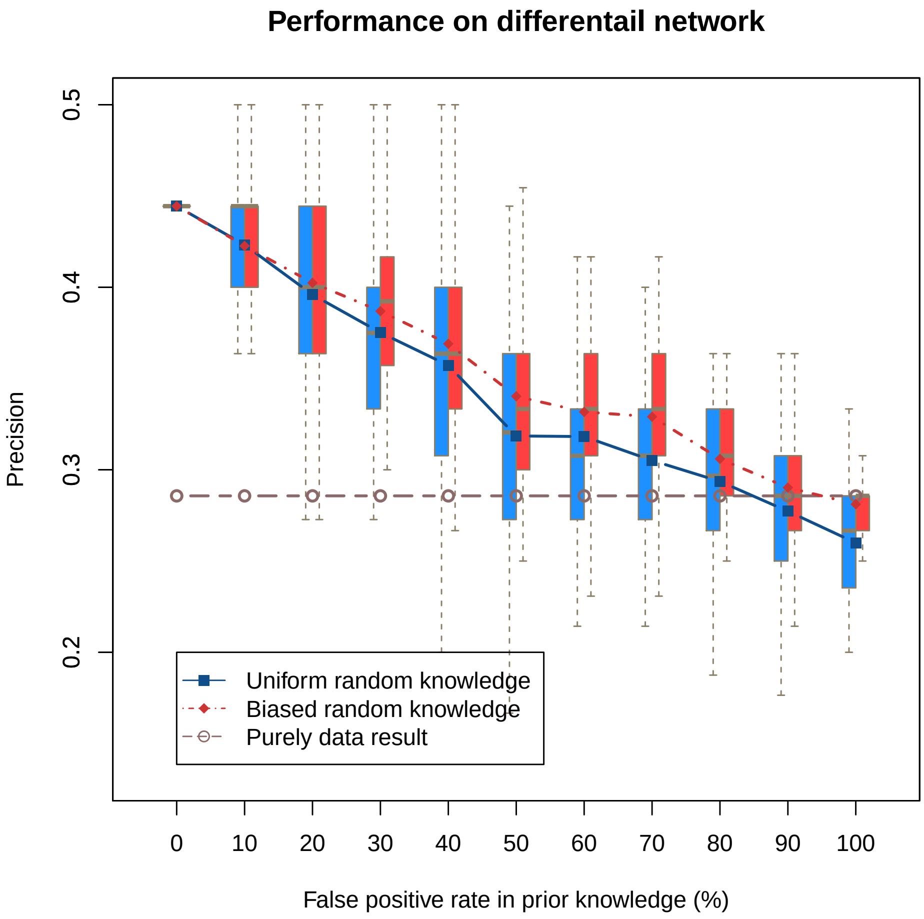

Figure S12: The effects of nonuniform random prior knowledge on inference precision of differential network.

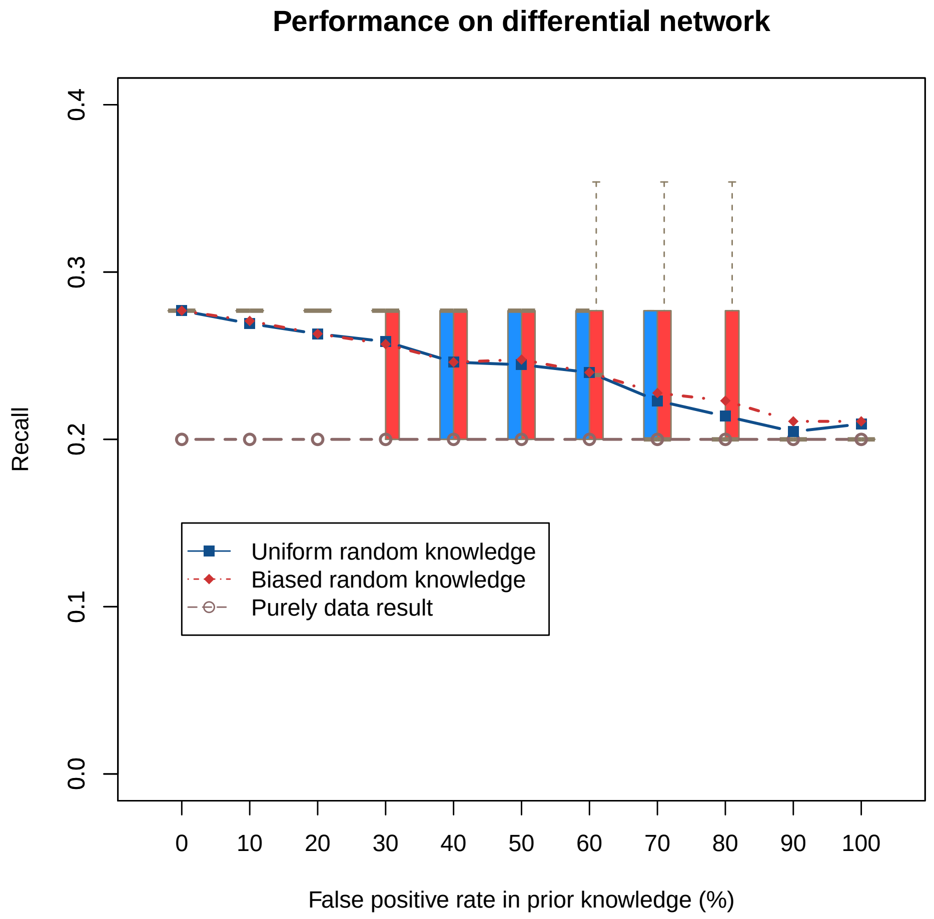

Figure S13: The effects of nonuniform random prior knowledge on inference recall of differential network.

S5.5 Effects of False Negatives in Prior Knowledge

Existing biological knowledge databases are mainly manually curated, which may suffer more from false negatives than false positives. However, the knowledge in databases are aggregated from general conditions. When they are used to guide inference of network under specific biological conditions, the inconsistency between knowledge and ground-truth become false positives. Unlike the assessment of databases quality, in knowledge incorpo-

rated inference, false positives are the major concerns as they directly affect the inference results, while false negatives which are the ground-truth not reflected by the knowledge do not affect the inference results.

To show this experimentally, we simulated the scenarios with different amount of false negatives in prior knowledge with fixed size of prior knowledge without the presence of false positives. When false positives present, increasing false negatives is equivalent to increasing false positives as we did in Figures 3 and 4, given fixed size of prior knowledge. Starting from all true knowledge, we gradually decrease the size of prior knowledge which is equivalent to adding in false negatives, until the size of prior knowledge is 0 and the inference purely relies on data. The results are shown in Figures S14 and S15. From the results of experiments with various false positive rate and false negative rate in prior knowledge, we can conclude that true positives in knowledge benefit the inference, false positives degrade the performance but our method controls it, and false negatives do not affect the inference.

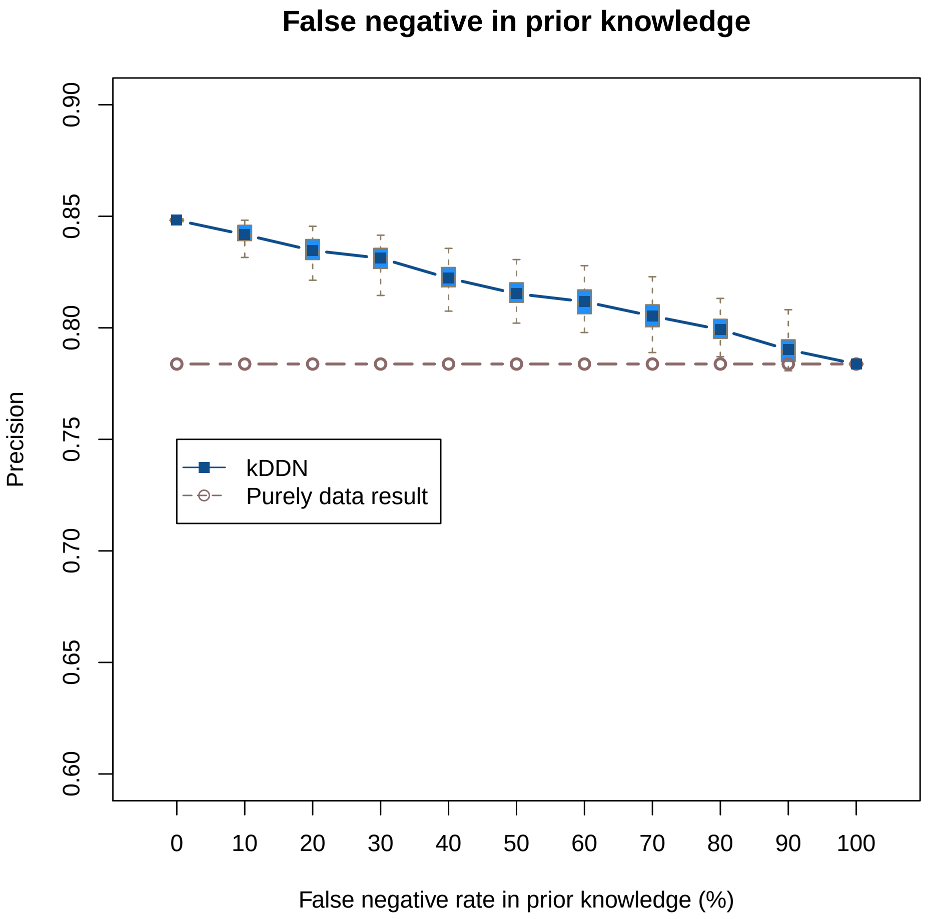

Figure S14: The effects of false negatives in prior knowledge on inference precision.

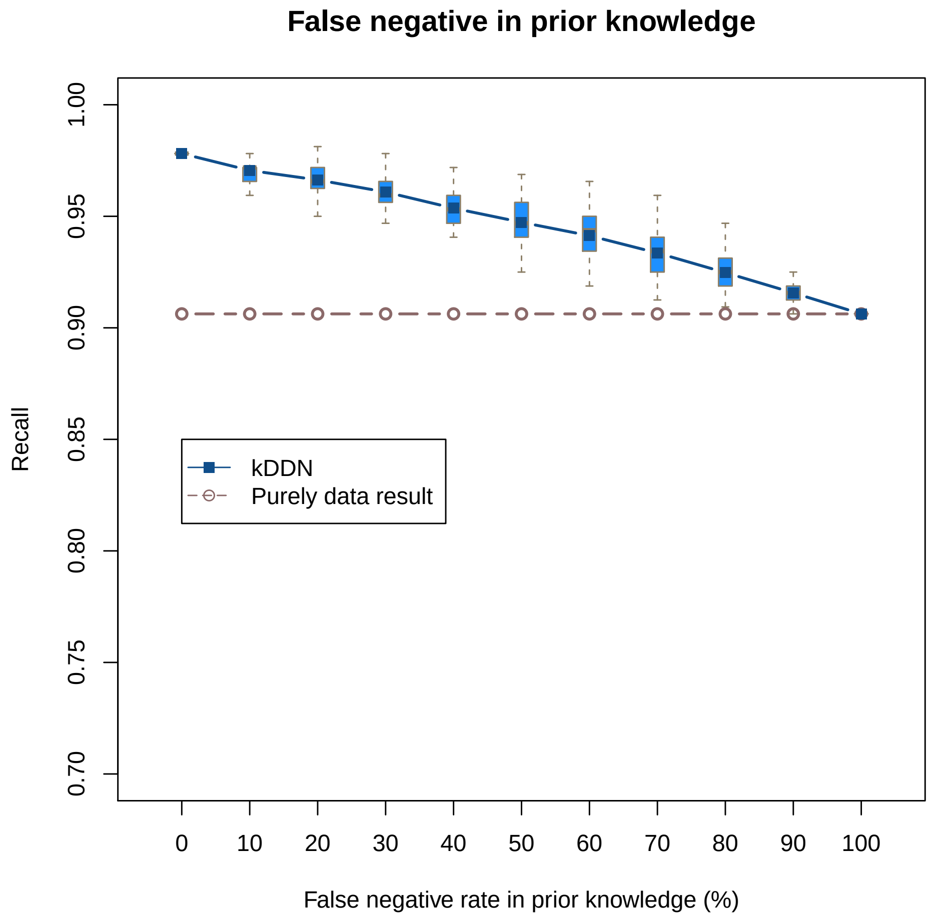

Figure S15: The effects of false negatives in prior knowledge on inference recall.

S5.6 Empirical Type I Error Rate for Simulated Data Sets Under the Null Hypothesis

We test the type I error rate of differential edge detection of kDDN using multiple simulation data sets under the null distribution (no differential edges between the two networks) to assess if the differential edges are identified at the right significance level. If the type I error rate is either too conservative or too liberal, the p-value fails to reflect the actual false positive rate and we cannot control how many false positives are detected by setting a

p-value based threshold (Chen *et al.*, 2011). Experiments show the average type I error rate under null distribution converges exactly to α under varied network sizes. This accuracy in p-value estimation gives stronger confidence in differential edge detection.

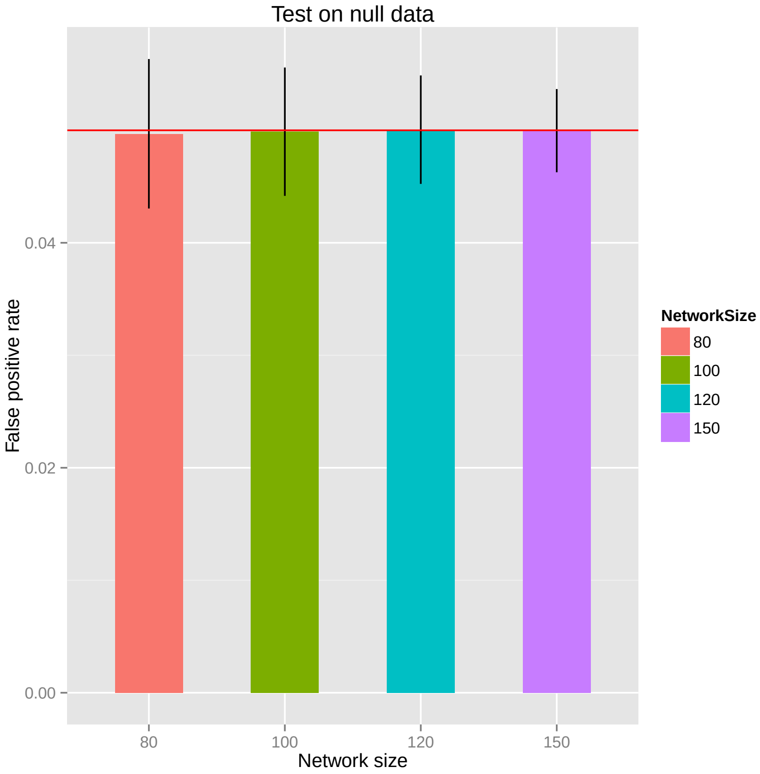

Figure S16: Empirical type I error rate (false positive rate) for simulated data sets under the null hypothesis under four different network sizes. The designed significance level $\alpha = 0.05$ is indicated by the red line. Across multiple runs, the average type I error rate is close to , which shows the differential edge detection is neither too conservative nor too liberal.

S5.7 Performance Comparison

The t-test results of performance comparison is detailed in Tables S1 and S2. Better results at 0.05 significance level are highlighted by bold font.

We plot the performance comparison results in Figure 5 on the plane of precision and

Table S1: T-test result of kDDN with peers in overall network learning performance.

Nodes in network	kDDN	csLearner	Meinshausen	DDN	Tesla
80	KDDN.dt	**4.19e-43**	**1.52e-56**	**6.26e-33**	**8.67e-08**
	KDDN.tk	**6.64e-46**	**3.86e-69**	**9.02e-45**	**2.08e-44**
	KDDN.fk	**2.06e-42**	**2.40e-52**	**7.58e-30**	**0.0039**
100	KDDN.dt	**1.81e-37**	**4.72e-53**	**2.11e-45**	**0.0499**
	KDDN.tk	**1.53e-41**	**2.79e-62**	**2.07e-50**	**4.21e-42**
	KDDN.fk	**6.40e-37**	**1.04e-45**	**7.19e-43**	0.9778
120	KDDN.dt	**2.20e-35**	**1.11e-99**	**3.99e-46**	**3.64e-06**
	KDDN.tk	**9.66e-37**	**4.86e-94**	**1.53e-47**	**8.74e-28**
	KDDN.fk	**5.49e-34**	**3.60e-94**	**1.19e-42**	0.99
140	KDDN.dt	**7.23e-34**	**1.63e-104**	**1.15e-47**	**1.35e-08**
	KDDN.tk	**3.45e-35**	**1.08e-101**	**6.83e-49**	**4.25e-15**
	KDDN.fk	**1.91e-32**	**2.37e-99**	**1.77e-44**	**0.0057**
160	KDDN.dt	**3.34e-46**	**1.75e-90**	**2.69e-51**	**2.56e-44**
	KDDN.tk	**4.31e-47**	**8.08e-83**	**5.32e-52**	**2.17e-47**
	KDDN.fk	**3.76e-45**	**4.55e-90**	**7.65e-50**	**4.22e-37**

Table S2: T-test result of kDDN with peers in differential network learning performance.

Nodes in network	kDDN	csLearner	Meinshausen	DDN	Tesla
80	KDDN.dt	**4.77e-12**	**3.45e-13**	0.2005	**0.0047**
80	KDDN.tk	**5.62e-16**	**1.34e-16**	**0.0075**	**1.72e-05**
80	KDDN.fk	**3.83e-12**	**2.20e-13**	0.2669	**0.0075**
100	KDDN.dt	**8.65e-16**	**3.41e-15**	**2.76e-05**	**0.0005**
100	KDDN.tk	**9.65e-18**	**8.55e-17**	**4.73e-08**	**1.90e-06**
100	KDDN.fk	**2.27e-14**	**6.65e-14**	**0.0001**	**0.0015**
120	KDDN.dt	**4.89e-40**	**3.95e-34**	**3.73e-25**	**2.08e-13**
120	KDDN.tk	**1.95e-41**	**1.31e-34**	**1.17e-28**	**9.33e-17**
120	KDDN.fk	**2.62e-39**	**2.46e-34**	**1.39e-22**	**4.39e-11**
140	KDDN.dt	**4.53e-35**	**9.76e-33**	**1.10e-24**	**1.01e-09**
140	KDDN.tk	**4.47e-36**	**4.67e-33**	**4.15e-27**	**4.41e-12**
140	KDDN.fk	**1.38e-34**	**3.15e-33**	**5.88e-22**	**3.24e-07**
160	KDDN.dt	**4.43e-41**	**4.20e-34**	**1.45e-32**	**2.05e-19**
160	KDDN.tk	**4.32e-46**	**4.28e-37**	**4.21e-38**	**2.64e-25**
160	KDDN.fk	**4.20e-40**	**1.14e-33**	**3.09e-29**	**4.53e-15**

recall, with background heatmap indicating F score ranging from 0 to 1, in Figures S17 and S18. Besides the same observation of F score performance in the paper, we also see that kDDN performs best in both precision and recall. Tesla performs the second. Meinshausen's method performs third in overall network but poor in differential network.

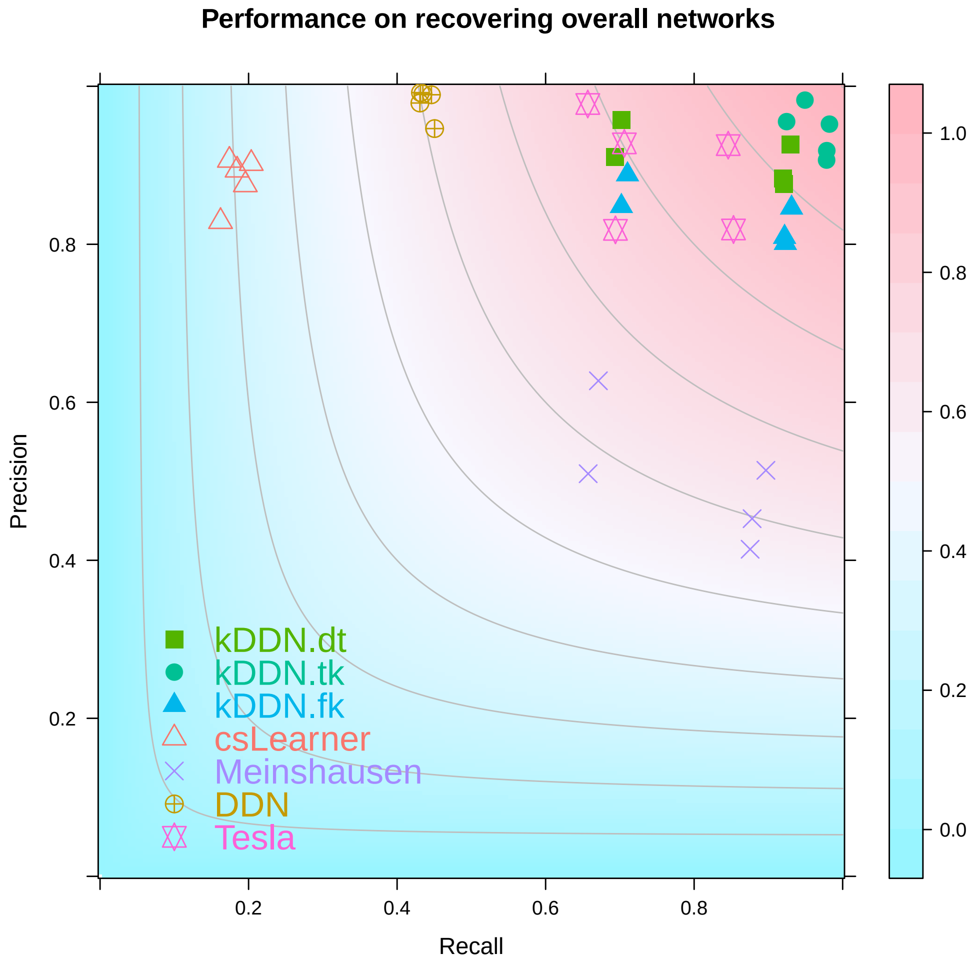

Figure S17: Performance of overall network recovery displayed on the plane of precision and recall with F score heatmap as background.

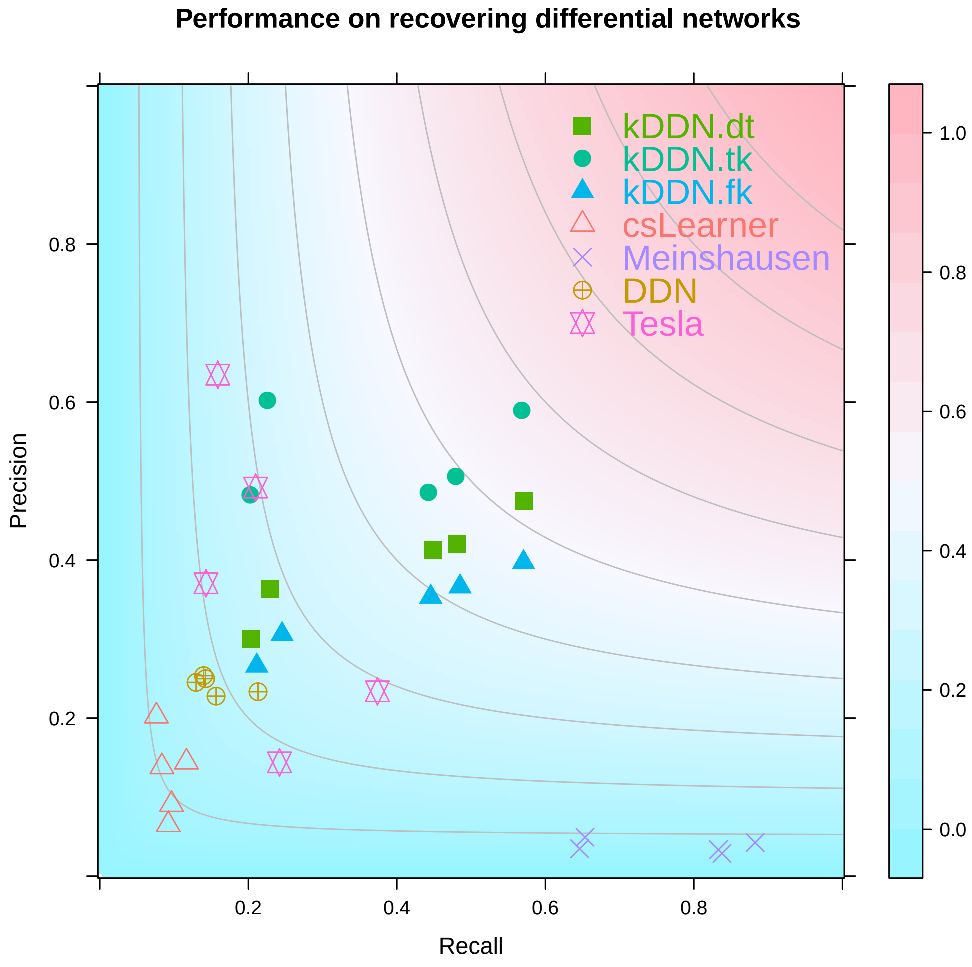

Figure S18: Performance of differential network recovery displayed on the plane of precision and recall with F score heatmap as background.

In addition to the performance comparison on networks with different sizes, we also compared kDDN with DDN on the simulation example used in (Zhang *et al.*, 2009). The example was generated by SynTren and the comparison is shown in Figure S19. The nodes are placed at the same relative positions. The ground truth network is shown in Figure S19(b), with 20 nodes. The black edges indicate common edges. Red and green edges are condition-specific edges. The result learned by DDN is shown in Figure S19(c), in which only differential edges are learned, and 3 edges are error. The network learned by kDDN

is shown in Figure S19(a). The number of erroneous edges is still 3 but 5 common edges are also identified. In this example the two methods achieved comparable performance in differential edge detection due to small network size, but the common edge identification can only be done by the proposed method.

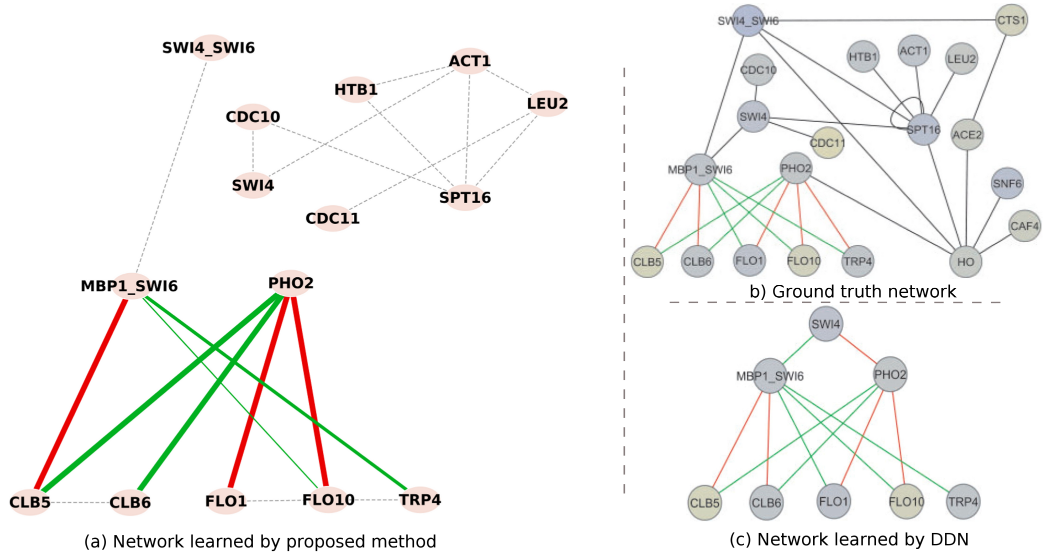

Figure S19: Comparison of results on simulation data generated by SynTren. (a) The result learned by the proposed method. (b) Ground truth network. (c) Network learned by DDN method.

S6 Additional Real Data Results

S6.1 Yeast and Breast Cancer Results with $\theta = 0$

We reported the experimental results on the stress/breast cancer datasets using the data-only method ($\theta = 0$, joint learning). On yeast oxidative stress response dataset, the network inferred from data-only experiment differs from that of data-knowledge integration by 14 differential edges, as shown in Figure S20 with differences highlighted. On the breast cancer dataset, the network inferred from data-only experiment differs from that of data-knowledge integration by 41 common and 1 differential edges between *CSF2RB* and *PIK3R5*.

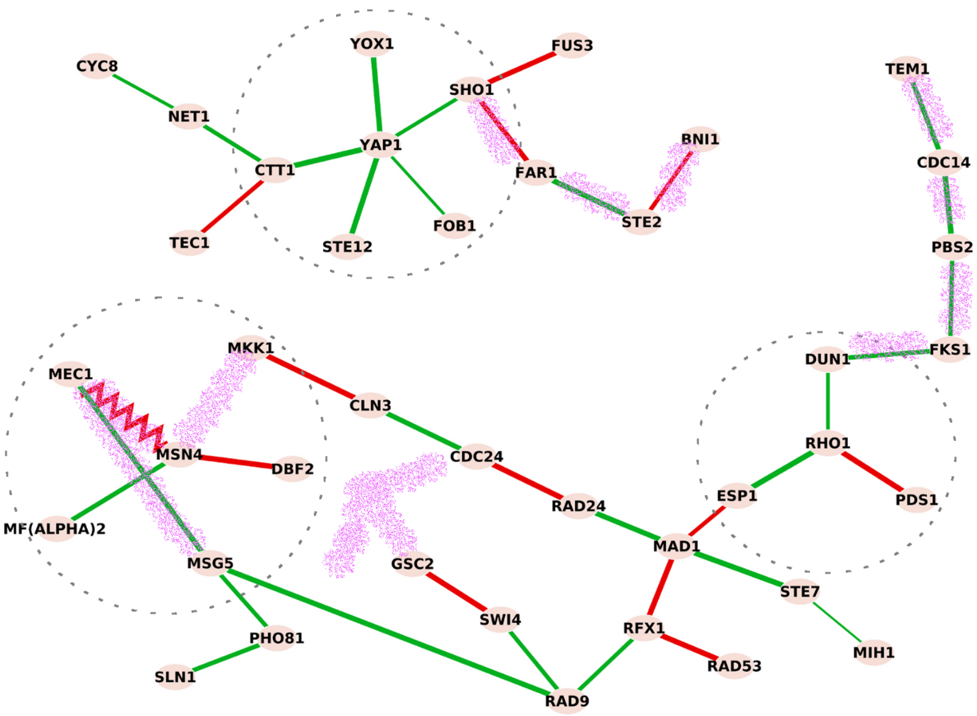

Figure S20: The differences of 14 differential edges between data-only result and knowledge incorporated result are highlighted.

S6.2 Robustness Analysis on Yeast Case Study

In the differential network identified, *Yap1*, *Rho1* and *Msn4* are at the center of the network. We used 100 bootstrap samples to evaluate whether this finding is robust. The degree of connections is a good indicator of the centrality of *Yap1*, *Rho1* and *Msn4*, which has a degree of 5, 3 and 5 in the network. In the bootstrap experiments we calculated the average degree of *Yap1*, *Rho1* and *Msn4*, which were 4.35, 2.98 and 5.06, with a standard deviation of 1.43, 1.33 and 1.53, respectively. The bootstrap degree showed that the genes are robustly identified as the “hubs” of the network. The robustness is important, but the sensitivity is also important to a biologist to provide hypothesis, which must be validated experimentally.

S6.3 A Case Study on Juvenile Dermatomyositis

We accessed a 125 patient muscle biopsy U133A mRNA profiling dataset containing 13 diagnostic groups of patients with specific muscle disorders (Bakay *et al.*, 2006). We applied kDDN to two of the largest groups: normal controls (NHM) with 18 samples and juvenile dermatomyositis (JDM) with 25 samples. Juvenile dermatomyositis is an autoimmune disorder of muscle in pediatric patients, and we have previously reported mRNA profiling of this disease in an earlier data set (Tezak *et al.*, 2002).

We queried two pathways that are known to be important in the pathophysiology of JDM using the KEGG database: apoptosis pathway (Figure S21), and T cell receptor signalling pathway (Figure S22).

The apoptosis pathway result illuminated important induction of apoptosis in JDM muscle, as shown by the strong upregulation of *Bax* and downregulation of *Bcl-2*, leading to a dramatic change in *Bax/Bcl-2* ratios associated with apoptosis (Rosse *et al.*, 1998). Apoptosis in JDM muscle has been previously reported (Zhao *et al.*, 2007). However, our result shows how co-regulation of key apoptosis regulatory proteins impinging on *Bax/Bcl-2* ratios is altered in normal control muscle *vs.* JDM muscle. For example, in normal muscle, *Bax* and its regulatory partner *TNFSF10* (TNF-related apoptosis-inducing ligand, also called *TRAIL*) are inversely correlated (red edge), yet in JDM they are directly correlated. *TNFSF10/TRAIL* is a ligand for apoptosis receptors, and the visualization suggests an abnormal positive feedback loop between *Bax* and *TNFSF10* that would be expected to be deleterious to myofiber survival. A similar situation is seen with *BCL2*, a key anti-apoptotic protein. In normal muscle, expression of *BCL2* and its regulatory protein *RIPK1* are inversely correlated. In JDM, they are simultaneously down-regulated, again suggestive of loss of negative regulatory loops, and promotion of apoptosis in JDM. *RIPK1* is a less well characterized protein, and the identification of its abnormal regulatory relationship with *BLC2* may point out new areas for further investigation.

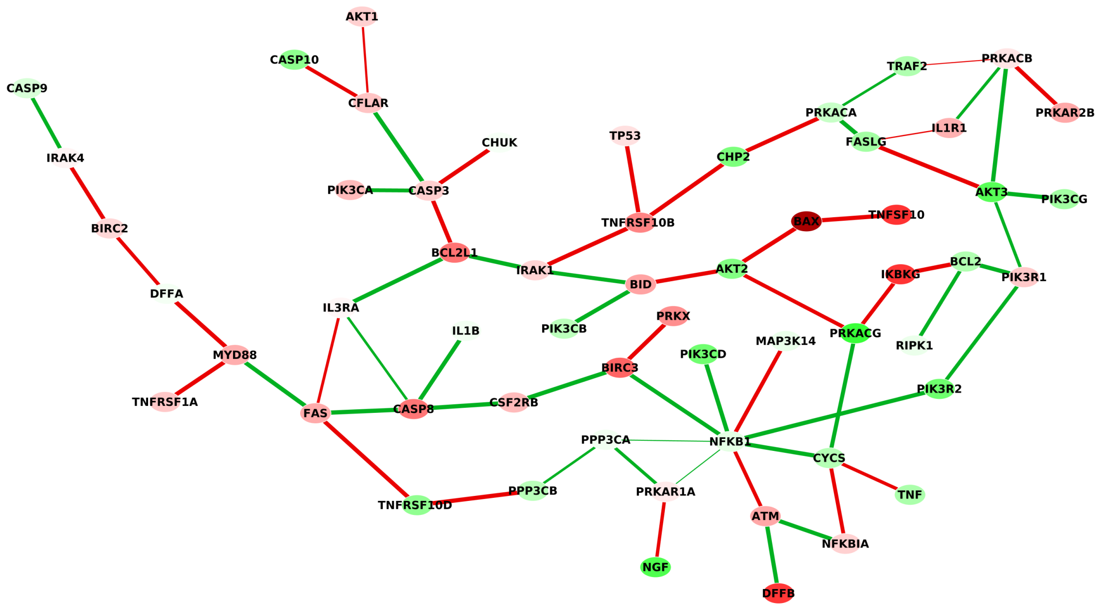

Figure S21: NHM *vs.* JDM in apoptosis pathway shows widespread evidence of loss of normal apoptotic regulatory control of apoptosis pathways in JDM. Red edges are in normal, and green edges are in JDM. Red nodes are up-regulated in JDM and green indicate down-regulation in JDM.

CD8+ T cells are a well-recognized inflammatory infiltrate in JDM muscle. When visualizing T cell receptor pathways via kDDN result, this is immediately apparent, with upregulation of CD8+ associated proteins. As normal skeletal muscle shows very few resident T cells, the visualization of differential network using kDDN provides fewer novel insights compared to the apoptosis example above. However, a novel and potentially important sub-network was detected by kDDN due to the role of NFAT proteins in mediating membrane signals to nuclear cellular reactions. *NFAT5* is shown by DDN visualization to be highly upregulated in JDM. *NFAT5* is known to respond to osmostic stress, relaying this signal to the nucleus in muscle and other tissues (Zhang *et al.*, 2003; Hernndez-Ochoa *et al.*, 2012). The connection to T cell pathways via *ZAP70* likely reflects an interaction between infiltrating T cells and pro-inflammatory myofibers, and points out a possible ischemic/osmotic pathway that may be an important contributor to JDM.

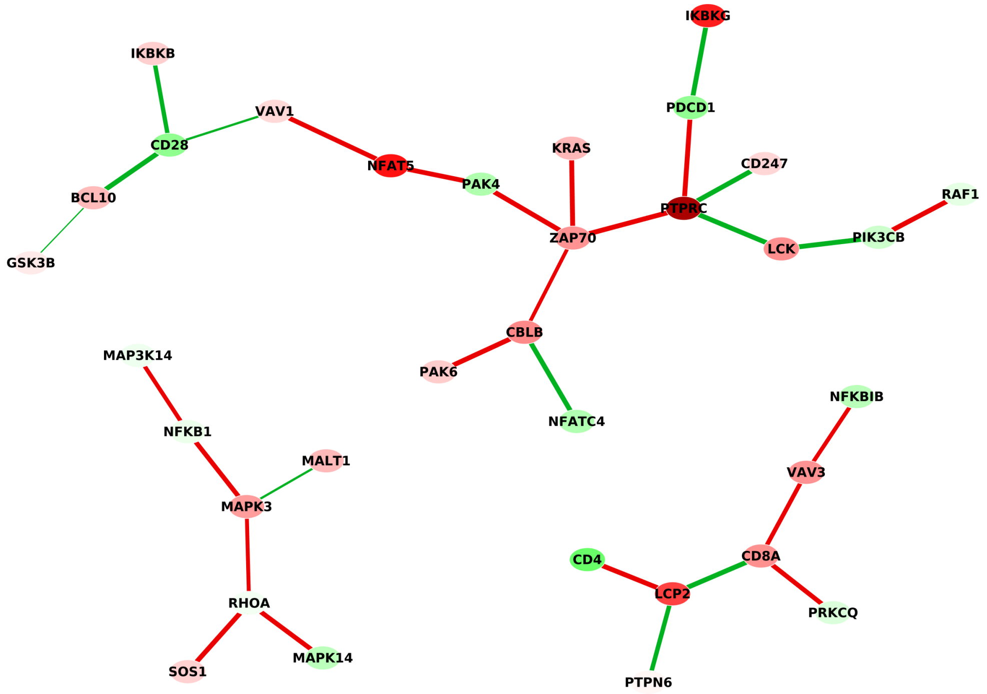

Figure S22: NHM *vs.* JDM in T cell receptor signalling pathway. Red edges are in normal, and green edges are in JDM. Red nodes are up-regulated in JDM and green indicate down-regulation in JDM.

S6.4 A Case Study on Transcription Factor Estrogen Receptor α Regulation

Nuclear receptor estrogen receptor alpha (ERα) controls the expression of target genes through either direct or tethered DNA binding. It is important to study the differential binding patterns of ERα under different conditions to understand the mechanisms of ERα binding. We used a public data set (Stender *et al.*, 2010) profiling the gene expression of wild type and ERα mutated cell lines to discover the different binding targets.

The wild type ERα is expected to regulate target gene expression via direct binding while the ERα muated regulation is expected to be accomplished via tethered DNA binding. We selected a set of genes from literature documented ERα targets (Klinge, 2001; Lin

et al., 2004), expression inferred possible targets (Stender *et al.*, 2010) and targets found in database (Klinge, 2001), including *ESR1*, *TFF1*, *EBAG9*, *CASP7*, *GREB1*, *SP1*, *JUN*, *FOSB*, *ATF*, *CEBPB*, *PITX1*, *GADD45A*, *TRIB1*, *SOX9* and *HBEGF*. Results are shown in Figure S23.

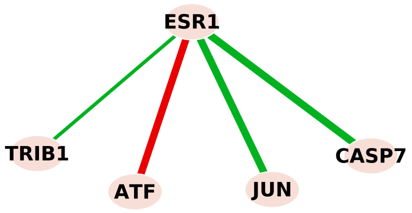

Figure S23: Wild type ERα *vs.* ERα mutated shows differential DNA binding patterns via direct and tethered binding. Red edges are in wild type, and green edges are in ERα muated.

The identified target binding relations pointed out the possible binding schemes as green - tethered and red - direct or tethered. *JUN* and *ATF* are known to be involved in tethered binding (Umayahara *et al.*, 1994; Kushner *et al.*, 2000), while *CASP7* is known as a direct target (Klinge, 2001; Lin *et al.*, 2004). These results demonstrated the ability of kDDN to work with transcription factor-target information and identify condition-specific transcription factor binding.

References

Bakay, M., Wang, Z., Melcon, G., Schiltz, L., Xuan, J., Zhao, P., Sartorelli, V., Seo, J., Pegoraro, E., Angelini, C., Shneiderman, B., Escolar, D., Chen, Y.-W., Winokur, S. T., Pachman, L. M., Fan, C., Mandler, R., Nevo, Y., Gordon, E., Zhu, Y., Dong, Y., Wang, Y., and Hoffman, E. P. (April 2006). Nuclear envelope dystrophies show a transcriptional fingerprint suggesting disruption of rbmyod pathways in muscle regeneration. *Brain*, **129**(4), 996–1013.

Banerjee, O., El Ghaoui, L., and d'Aspremont, A. (2008). Model selection through sparse

maximum likelihood estimation for multivariate gaussian or binary data. *J. Mach. Learn. Res.*, **9**, 485–516.

Chen, L., Yu, G., Langefeld, C., Miller, D., Guy, R., Raghuram, J., Yuan, X., Herrington, D., and Wang, Y. (2011). Comparative analysis of methods for detecting interacting loci. *BMC Genomics*, **12**(1), 344.

Friedman, J., Hastie, T., and Tibshirani, R. (2008). Sparse inverse covariance estimation with the graphical lasso. *Biostatistics*, **9**(3), 432–441.

Genz, A. and Bretz, F. (2009). *Computation of Multivariate Normal and t Probabilities*. Lecture Notes in Statistics. Springer-Verlag, Heidelberg.

Genz, A., Bretz, F., Miwa, T., Mi, X., Leisch, F., Scheipl, F., and Hothorn, T. (2012). *mvtnorm: Multivariate Normal and t Distributions*. R package version 0.9-9994.

Hernndez-Ochoa, E. O., Robison, P., Contreras, M., Shen, T., Zhao, Z., and Schneider, M. F. (2012). Elevated extracellular glucose and uncontrolled type 1 diabetes enhance nfat5 signaling and disrupt the transverse tubular network in mouse skeletal muscle. *Experimental Biology and Medicine*, **237**(9), 1068–1083.

Klinge, C. M. (2001). Estrogen receptor interaction with estrogen response elements. *Nucleic Acids Research*, **29**(14), 2905–2919.

Kushner, P. J., Agard, D. A., Greene, G. L., Scanlan, T. S., Shiau, A. K., Uht, R. M., and Webb, P. (2000). Estrogen receptor pathways to ap-1. *The Journal of Steroid Biochemistry and Molecular Biology*, **74**(5), 311 – 317.

Lin, C.-Y., Strom, A., Vega, V., Li Kong, S., Li Yeo, A., Thomsen, J., Chan, W., Doray, B., Bangarusamy, D., Ramasamy, A., Vergara, L., Tang, S., Chong, A., Bajic, V., Miller, L., Gustafsson, J.-A., and Liu, E. (2004). Discovery of estrogen receptor alpha target genes and response elements in breast tumor cells. *Genome Biology*, **5**(9), R66.

Meinshausen, N. and Bühlmann, P. (2006). High-dimensional graphs and variable selection with the lasso. *The Annals of Statistics*, **34**(3), 1436–1462.

Rosse, T., Olivier, R., Monney, L., Rager, M., Conus, S., Fellay, I., Jansen, B., and Borner, C. (1998). Bcl-2 prolongs cell survival after bax-induced release of cytochrome c. *Nature*, **391**(6666), 496–499.

Stender, J. D., Kim, K., Charn, T. H., Komm, B., Chang, K. C. N., Kraus, W. L., Benner, C., Glass, C. K., and Katzenellenbogen, B. S. (2010). Genome-wide analysis of estrogen receptor dna binding and tethering mechanisms identifies runx1 as a novel tethering factor in receptor-mediated transcriptional activation. *Molecular and Cellular Biology*, **30**(16), 3943–3955.

Tezak, Z., Hoffman, E. P., Lutz, J. L., Fedczyna, T. O., Stephan, D., Bremer, E. G., Krasnoselska-Riz, I., Kumar, A., and Pachman, L. M. (2002). Gene expression profiling in dqa1*0501+ children with untreated dermatomyositis: A novel model of pathogenesis. *The Journal of Immunology*, **168**(8), 4154–4163.

Umayahara, Y., Kawamori, R., Watada, H., Imano, E., Iwama, N., Morishima, T., Yamasaki, Y., Kajimoto, Y., and Kamada, T. (1994). Estrogen regulation of the insulin-like growth factor i gene transcription involves an ap-1 enhancer. *Journal of Biological Chemistry*, **269**(23), 16433–42.

Zhang, B. and Wang, Y. (2010). Learning structural changes of gaussian graphical models in controlled experiments. In *Conference on Uncertainty in Artificial Intelligence (UAI 2010)*.

Zhang, B., Li, H., Riggins, R. B., Zhan, M., Xuan, J., Zhang, Z., Hoffman, E. P., Clarke, R., and Wang, Y. (2009). Differential dependency network analysis to identify condition-specific topological changes in biological networks. *Bioinformatics*, **25**(4), 526–532.

Zhang, Z., Ferraris, J. D., Brooks, H. L., Brisc, I., and Burg, M. B. (2003). Expression of

osmotic stress-related genes in tissues of normal and hyposmotic rats. *American Journal of Physiology - Renal Physiology*, **285**(4), F688–F693.

Zhao, Y., Fedczyna, T. O., McVicker, V., Caliendo, J., Li, H., and Pachman, L. M. (2007). Apoptosis in the skeletal muscle of untreated children with juvenile dermatomyositis: Impact of duration of untreated disease. *Clinical Immunology*, **125**(2), 165 – 172.
